# Supplementary material for: HCMV pUL135 Remodels the Actin Cytoskeleton to Impair Immune Recognition of Infected Cells
Source: Cell Host Microbe. 2014 Aug 13;16(2):201–14. doi: 10.1016/j.chom.2014.07.005 (PMC4150922; doi:10.1016/j.chom.2014.07.005)
Supplement: Document S1. Supplemental Experimental Procedures, Figures S1–S7, and Tables S1 and S2 [file mmc1.pdf]

Cell Host & Microbe, Volume *16*

Supplemental Information

HCMV pUL135 Remodels the Actin Cytoskeleton to Impair Immune Recognition of Infected Cells

Richard J. Stanton, Virginie Prod'homme, Marco A. Purbhoo, Melanie Moore, Rebecca J. Aicheler, Marcus Heinzmann, Susanne M. Bailer, Jürgen Haas, Robin Antrobus, Michael P. Weekes, Paul J. Lehner, B. Vojtesek, Kelly L. Miners, Stephen Man, Gavin S. Wilkie, Andrew J. Davison, Eddie C.Y. Wang, Peter Tomasec, and Gavin W.G. Wilkinson

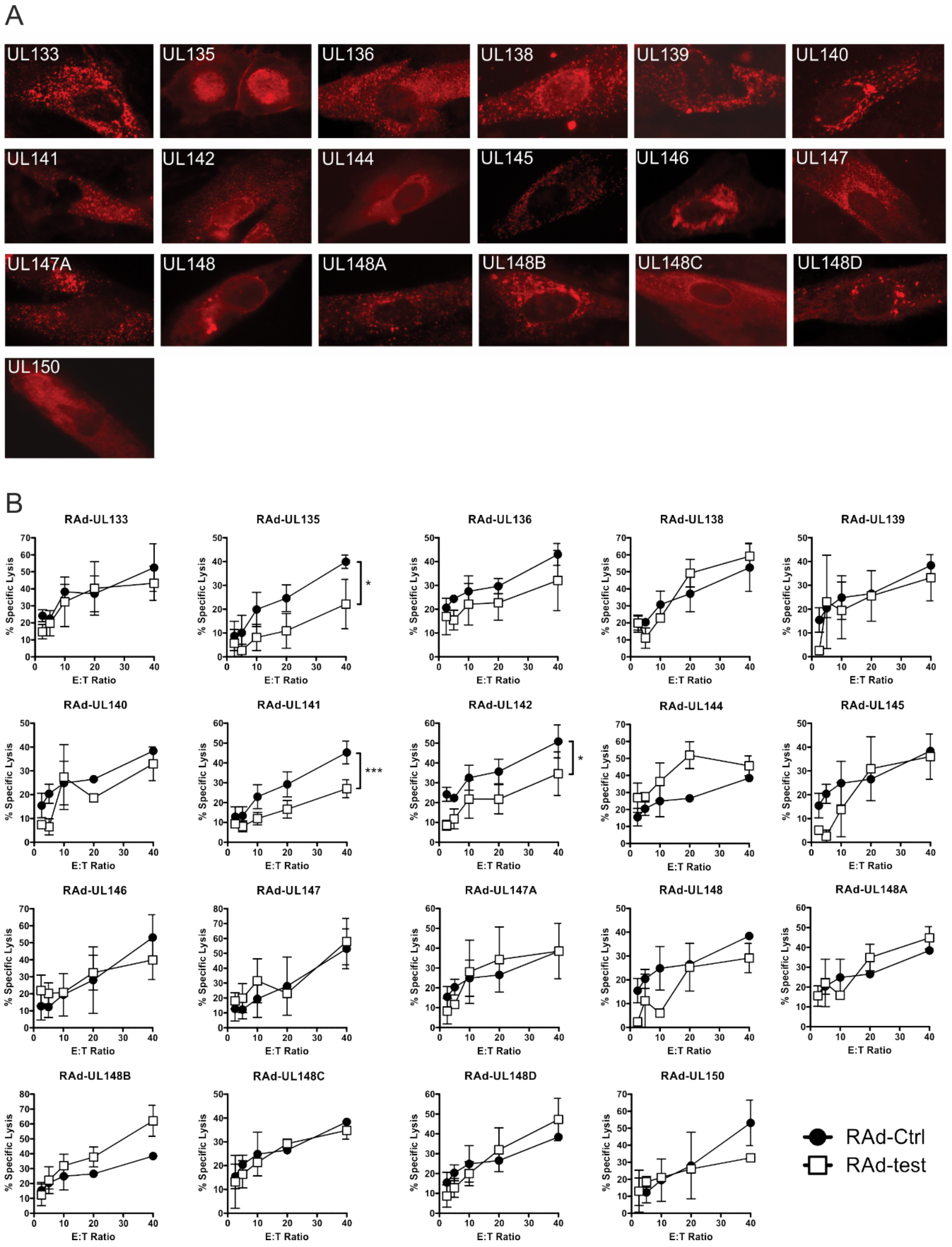

Figure S1, related to Figure 1. Expression, and ability of UL/*b'* proteins to protect from NK cell lysis. (A) HFFF-hCAR were infected with a RAd expressing one of the 19 UL/*b'* genes. 72 h post-infection expression of each gene was tested by immunofluorescence using a C-terminal tag engineered onto each protein. (B) Cytotoxicity assays were set up with the NKL clone against HFFF-hCAR infected with RAd vectors expressing each of the 19 UL/*b'* genes

(RAd-test) or empty vector control (Rad-Ctrl). Assays were performed 72 h after infection. Results are mean ± SD of triplicate samples (Two-Way ANOVA test: * $p < 0.05$, *** $p < 0.001$).

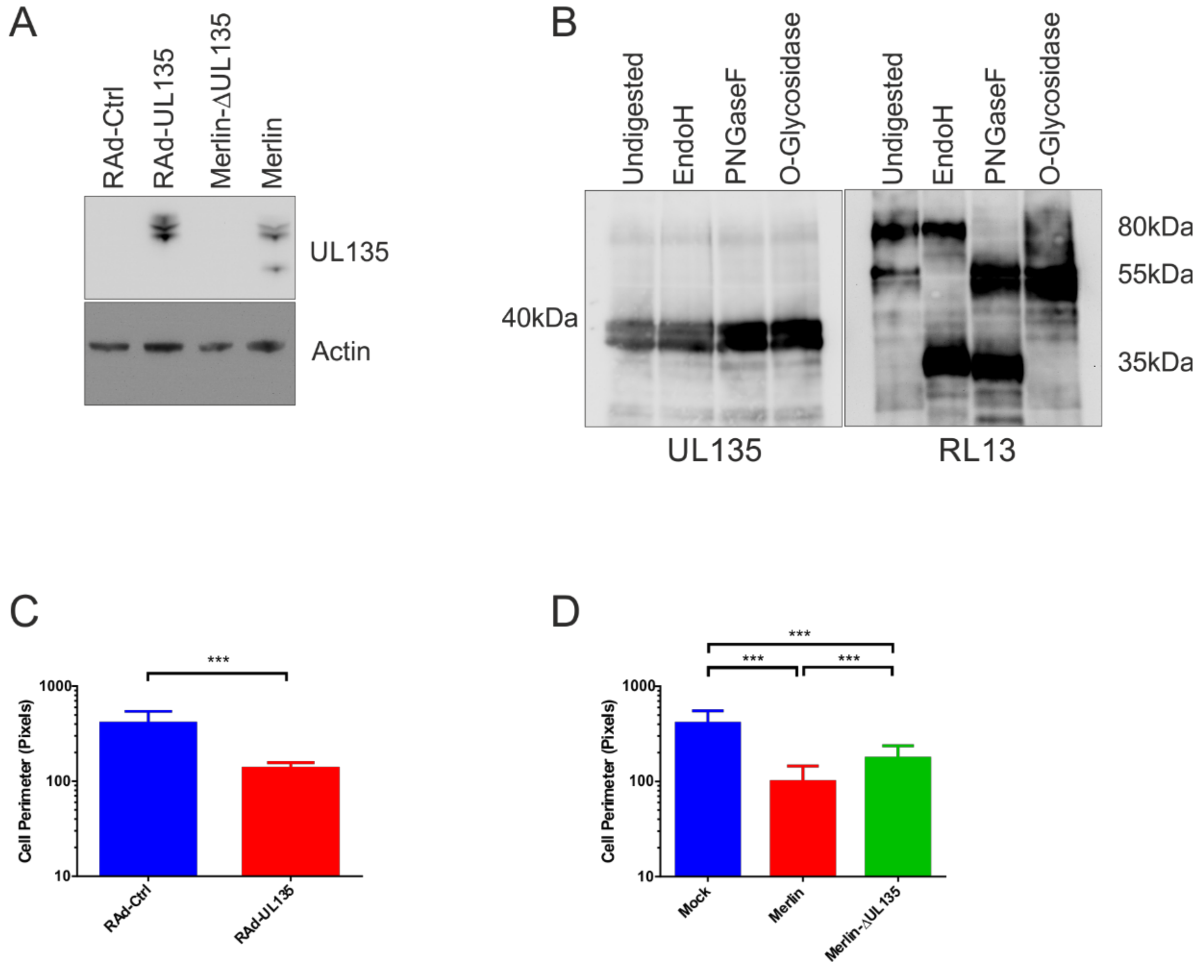

Figure S2, Related to Figure 2. Expression of pUL135. HFFF were infected with Merlin or MerlinΔUL135, and HFFF-hCAR were infected with RAd-Ctrl or RAd-UL135. (A) 48 h post infection samples were separated by SDS-PAGE and western blot performed for the indicated proteins. (B) Prior to SDS-PAGE, samples were deglycosylated with either EndoH, PNGaseF or O-Glycosidase. gpRL13 has both N- and O-linked sugars and therefore provides a positive control for the activity of the enzymes. (C-D) 48 h post infection, the perimeter of cells was calculated using imageJ. Error bars show SD. Unpaired t-test (C) or one-way ANOVA (D): *** $p < 0.001$.

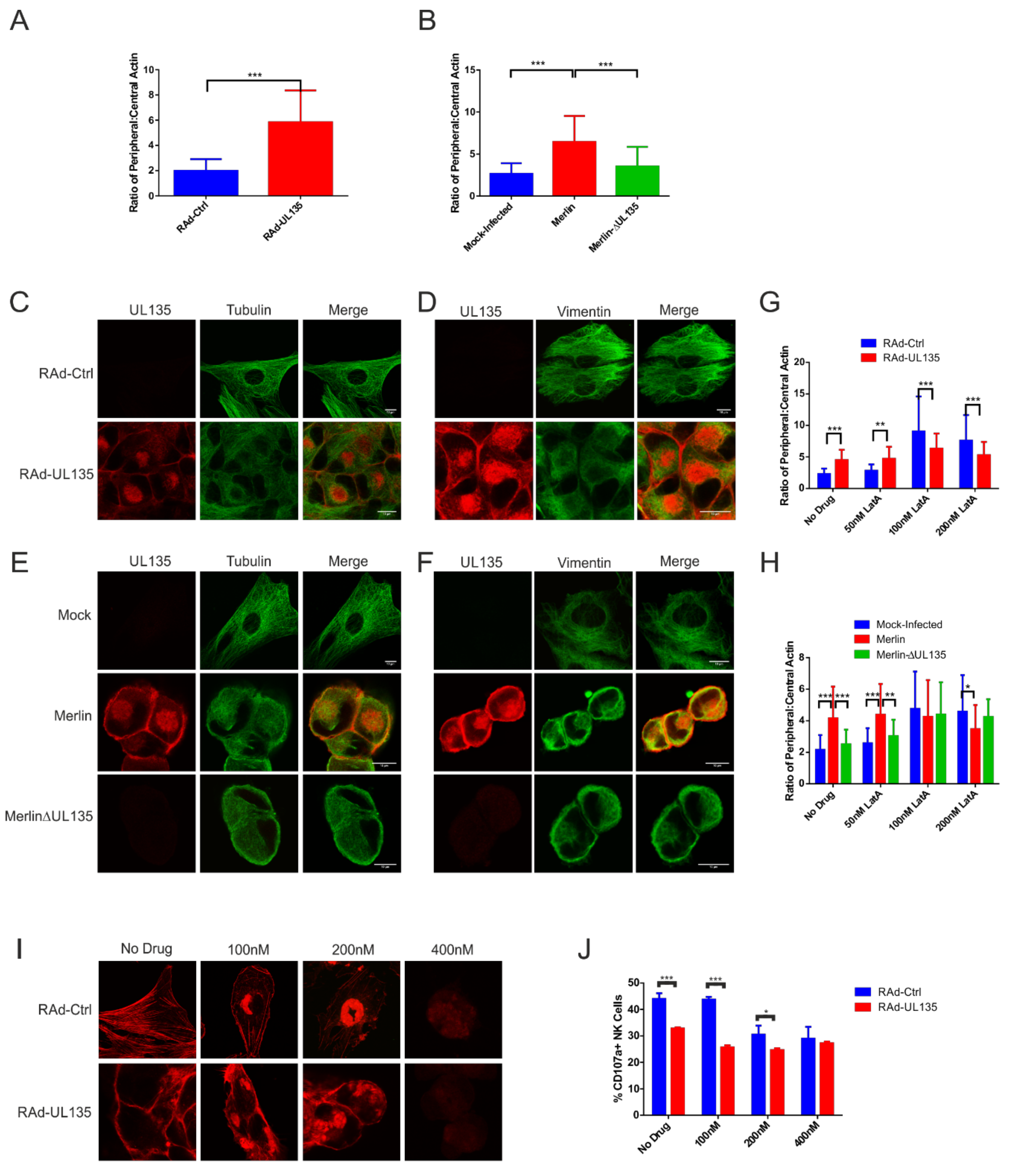

Figure S3, Related to Figure 3. .Effect of pUL135 on the cytoskeleton. HFFF were mock infected, or infected with Merlin or MerlinΔUL135 as indicated. HFFF-hCAR were infected with RAd-Ctrl or RAd-UL135 as indicated. (A) 48 h post infection, actin was stained with phalloidin, and the ratio of actin staining intensity at the cell periphery to staining in the center of the cell calculated. (C-F) 48 h post-infection, samples were stained for UL135 (V5 antibody) and tubulin (C, E) or vimentin (D, F). (G-H) Following infection, cells were treated with latrunculinA at the indicated doses. 48 h post infection, cells were fixed and stained with

phalloidin and the ratio of actin staining intensity at the cell periphery to staining in the center of the cell calculated. (I-J) Following infection, Jasplakinolide was added at the indicated concentrations. 48 hours later, cells were fixed and stained with phalloidin-AF594 (I) or NK degranulation assays were performed with IFNα stimulated PBMC (J). Error bars show SD. Unpaired t-test (A), one-way ANOVA (B), two-way ANOVA (G, H, J): * $p < 0.05$, ** $p<0.01$, *** $p < 0.001$.

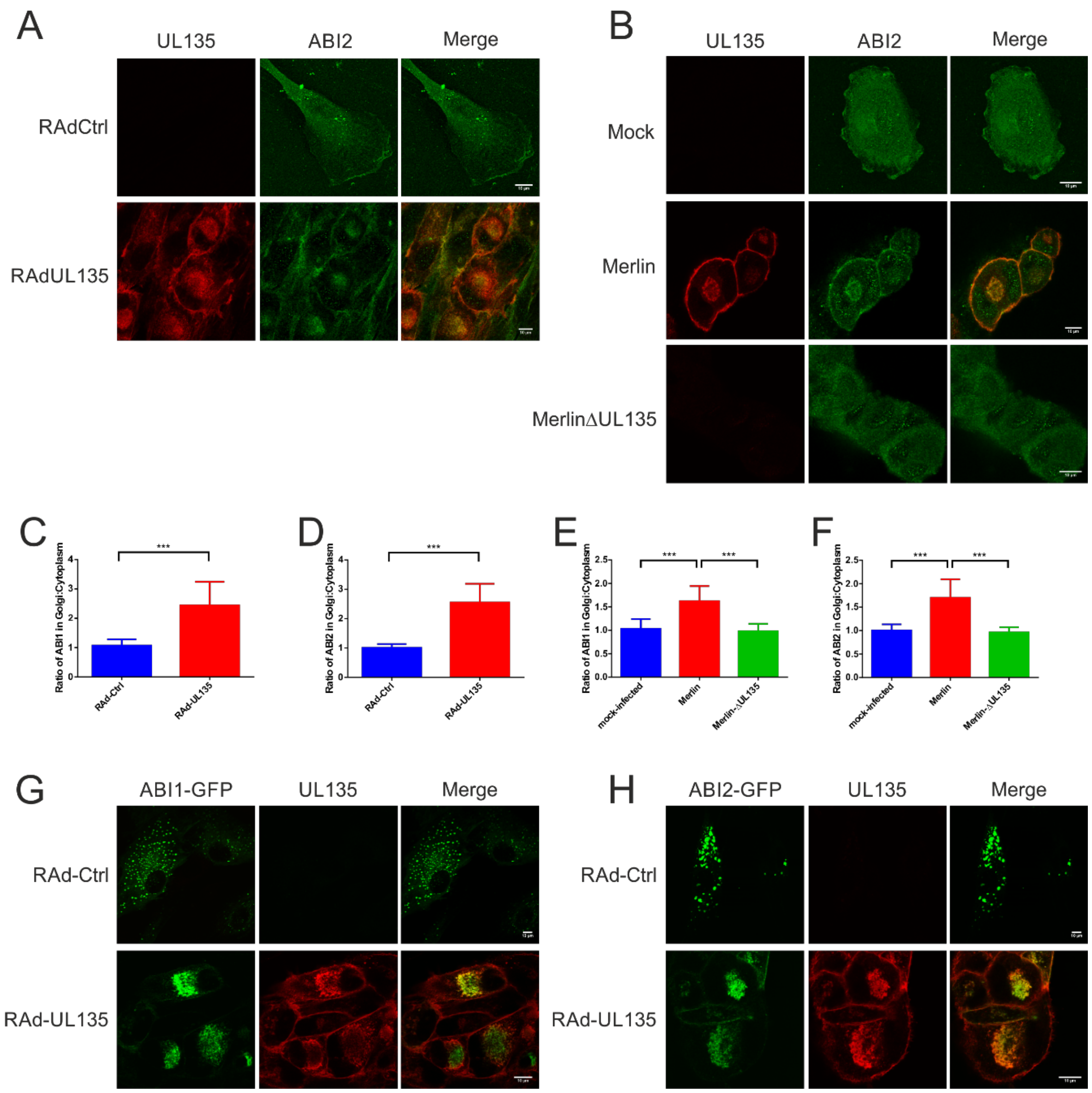

Figure S4, Related to Figure 4. pUL135 interacts with members of the WRC. HFFF-hCAR were infected with RAd-UL135 or RAd-Ctrl (A, C, D, H), and ABI1-GFP (G) or ABI2-GFP (H). HFFF were infected with HCMV strain Merlin, MerlinΔUL135 or mock infected (B, E, F). 48 h post infection samples were fixed and stained for UL135 (V5 antibody), giantin (a marker of the golgi) (C-F) and ABI1 (C, E) or ABI2 (A, B, D, F). (C-F) The amount of ABI1 (C/E) or ABI2 (D/F) that trafficked to the golgi was calculated as the mean fluorescence intensity of ABI1/2 that co-localised with the golgi, divided by the mean fluorescence intensity of ABI1/2 found in the cytoplasm. Error bars show SD. Unpaired t-test (C, D), one-way ANOVA (E, F):* $p < 0.05$, ** $p < 0.01$, *** $p < 0.001$)

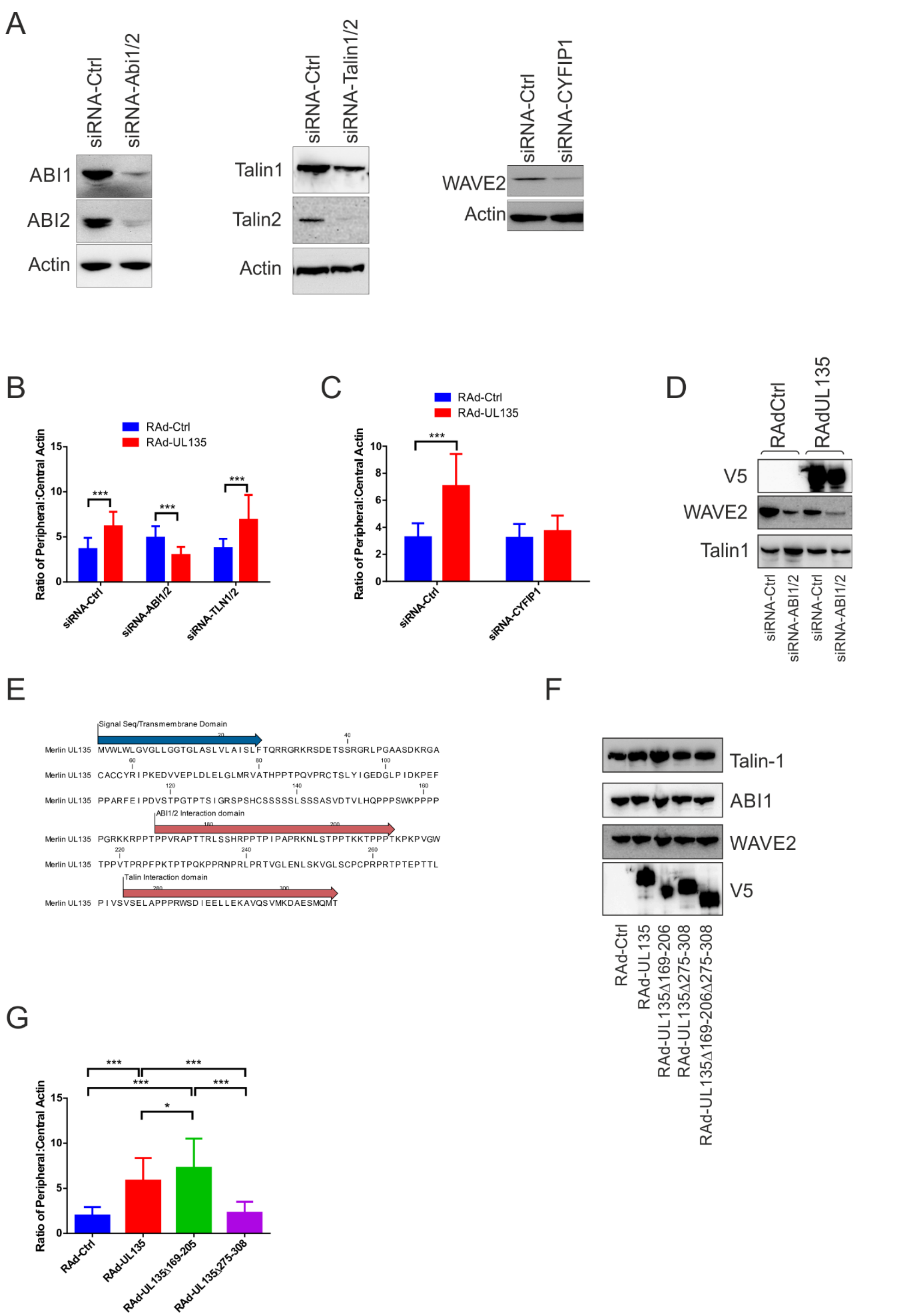

Figure S5, Related to Figure 5. The interaction of UL135 with the WRC results in actin remodelling. (A) Cells were transfected with the indicated siRNA. 72 h post-transfection,

proteins were separated by SDS-PAGE and western blot performed for indicated proteins. Note that for CYFIP1, no antibody was available to detect CYFIP1 in cell lysates, so WAVE2 levels were used as a surrogate marker. (B-D) 24 h after transfection of siRNA, HFFF-hCAR were infected with RAd-Ctrl or RAd-UL135. (B, C) 48 h PI, cells were fixed and stained with phalloidin, then the ratio of staining intensity at the cell periphery to staining in the center of the cell calculated. (D, F) shows input lysates as used in immunoprecipitations (Fig 5E, F). (E) The sequence of the pUL135 is depicted, along with the regions which, which deleted, abrogated the interaction with either the WRC, or Talin. Both a signal sequence (aa 1-22) and transmembrane domain (aa 4-26) are predicted at the N-terminus, indicative of a signal anchor peptide. (G) HFFF-hCAR were infected with the indicated RAd vectors, then 48 h PI, cells were fixed and stained with phalloidin, and the ratio of staining intensity at the cell periphery to staining in the center of the cell calculated. Error bars show SD. One-way ANOVA (G) or two-way ANOVA (B, C): * $p < 0.05$, ** $p < 0.01$, *** $p < 0.001$.

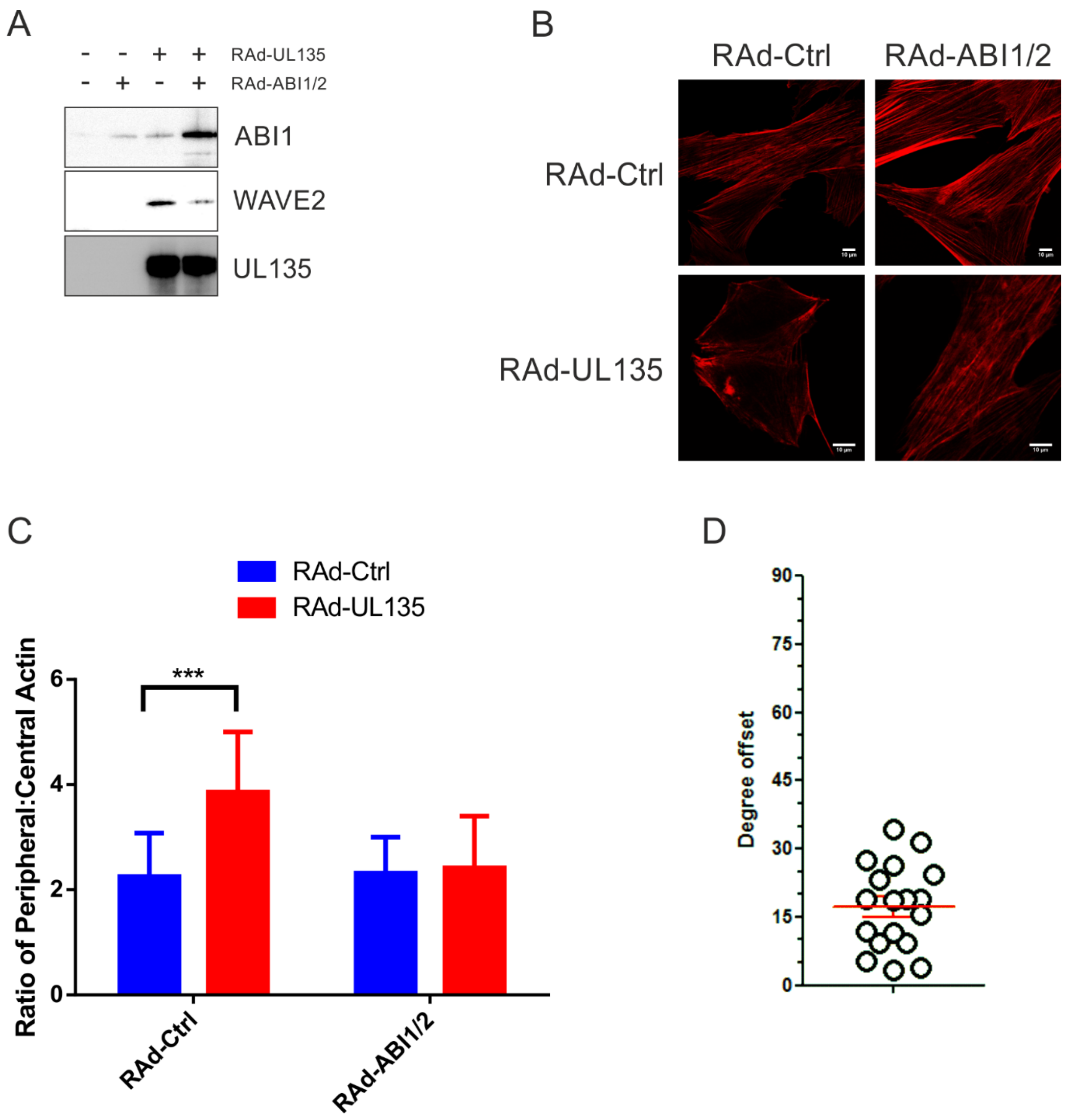

Figure S6, Related to Figure 6. The interaction of pUL135 with the WRC is required for inhibition of NK cells. (A-C) HF-hCAR were infected with either RAd-Ctrl or RAd-UL135, along with either RAd-Ctrl or RAd-ABI1 and RAd-ABI2 together. (A) 48 h post-infection proteins were immunoprecipitated using the V5 tag of UL135, run on a SDS-PAGE gel and western blotted before being stained for the indicated proteins. (B) 48 h post-infection cells were fixed and actin was stained with phalloidin, and (C) the ratio of actin in the periphery of the cell with that in the centre of the cell was calculated. Error bars show SD. Two-way ANOVA test, *** $p < 0.001$. (D) Directionality analysis was performed to determine the preferred orientation of the actin cytoskeleton in the HFF target cell and that of the F-actin in

the NKL cell within individual immune synapses. The plot shows the degree offset between the preferred actin orientation in the NKL cell and that of its target cell, with 0° indicating perfect alignment and 90° indicating a perpendicular alignment. Error bars show SEM. If there were there no correlation between F-actin in the NK synapse and F-actin in the target cell, the values would be equally spread between 0° and 90°. Instead the values cluster, with the NK synapse offset from the actin in the target cell by 15° on average.

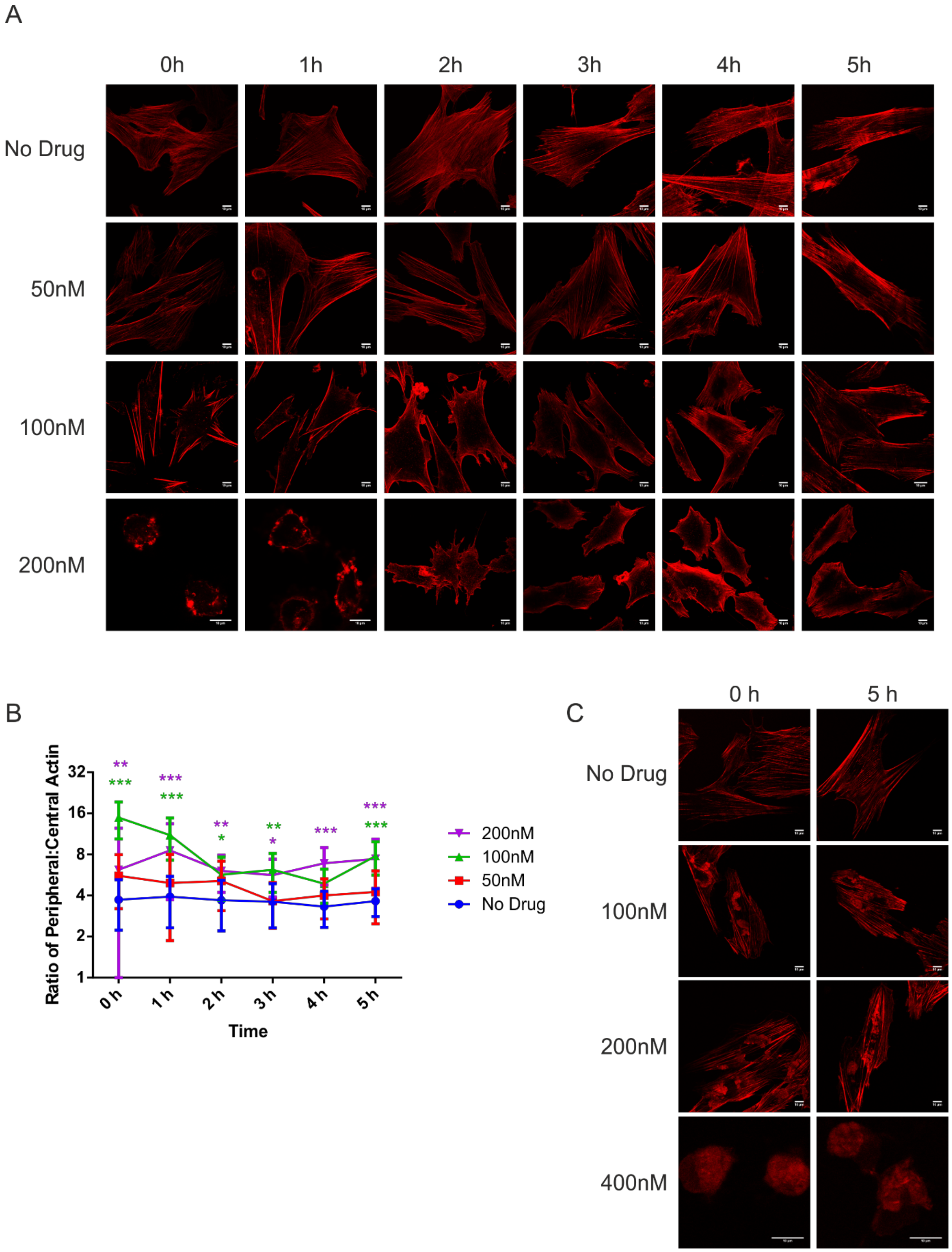

Figure S7, Related to Experimental Procedures. Recovery of actin during the timecourse of a CD107a assay following latrunculin A or jasplakinolide treatment. HFFF-hCAR were treated with LatA (A) or Jasplakinolide (C) for 48 hours, then the drug removed and replaced with RPMI. Cells were allowed to recover for the indicated times, then cells were fixed and

stained with Phalloidin-594. (B) The ratio of central:peripheral actin for the images in (A) were also quantitated. Error bars show SD. Two-way ANOVA, compared to 'No Drug': * $p < 0.05$, ** $p < 0.01$, *** $p < 0.001$.

Supplementary Table 1, related to table 4 – Proteins with cell surface expression not modulated by UL135.

NK Receptor Ligands
MICA-B, ULBP1-5, Nectin 1-2, CD47, CD48, CD66a (CEACAM-1), CD155 (PVR), CD324 (E-Cadherin), Necl2 (IGSF4), NTB-A (SLAMF6), CRACC (SLAMF7), HLA A, B, C, E.
**Adhesion Proteins**
CD2, CD11b, CD22, CD29 (integrin β1), CD31, CD34, CD36, CD41, CD42b, CD43, CD44, CD49a (integrin α1), CD49c (integrin α3), CD49d (integrin α4), CD49e (integrin α5), CD49f (integrin α6), CD50, CD51/CD61 (integrin αvβ3), CD54 (ICAM-1), CD55 (DAF), CD56, CD58 (LFA-3), CD99, CD99R, CD147, CD162.

HFFF-hCAR were infected for 72 h with a replication-deficient adenovirus expressing UL135 or a control adenovirus. Surface stainings were done with specific antibodies and isotype controls. Cells were analyzed by flow cytometry.

Supplementary Table 2, related to Fig. 4 – SILAC immunoprecipitation of UL135 interacting proteins

Protein Names	Uniprot	Log_2 H/L	H/L Sig A	Ratio Count	Unique peptides
NAP-1	Q9Y2A7	-4.620175	8.05E-07	15	8
Sra-1/PIR121	Q7L576	-4.549283	1.20E-06	14	10
UL135	F5HAQ7	-4.405956	2.63E-06	50	10
Talin-1	Q9Y490	-3.766976	6.50E-05	39	23
WAVE-2	Q9Y6W5	-3.696789	8.98E-05	2	3

Identified proteins are shown with corresponding UniProt accession number. SILAC ratios are shown as log_2 non-normalised values with associated significance A values as calculated in Persues v.1.1.1.13. SILAC ratios were calculated on the basis of all identified razor and unique peptides (ratio count) with the number of unique peptides identified for each protein indicated.

Supplementary Experimental Procedures

Generation of recombinant HCMV

To generate MerlinΔUL135 a cassette comprising ampicillin resistance, lacZ and SacB was recombineered in place of nt 189772-189086 (accession no. NC_006273) using primers 5’-CGGTGCGCGGCAGTCTCGGATTCCGCGGTGGCTTTTGTGGCGTCGGCGTTT TCGGGAAGGGCCTGGGCGTCACCGGCGCCTGTGACGGAAGATCACTTCG-3’ and 5’-CGGCCCTTCCCGACACGGAGTTTGAGATTCCAAGCAGGAGAGAAGATCATGGTG TGGCTGTGGCTCGGCGTCGGGCTCCTGAGGTTCTTATGGCTCTTG-3’. The cassette was removed using primer 5’-AGCCCGACGGTGCGCGGCAGTCTCGGATTCCGCGGTGGCTTT TGTGGCGTGATCTTCTCTCCTGCTTGGAATCTCAAACTCCGTGTCGGGAAGGGCC GGT-3’, deleting the first 687bp of the *UL135* open reading frame. MerlinΔUL141 has been described (Prod'homme et al., 2010). MerlinΔUL135/ΔUL141 BAC was generated from MerlinΔUL141 using the same strategy. To optimize detection in the context of a HCMV strain Merlin infection, a sequence providing a C-terminal V5 tag was inserted into *UL135.* The cassette comprising ampicillin resistance, lacZ and SacB was recombineered at the end of *UL135* using primers 5’-TCTCTTAGCTGCGGTGAAAAAGAGGGAAGGCGTGTGCTGCTATACAACTGTACA ACGGACGCGCTCGCTGTTTCGGTCCCTGTGACGGAAGATCACTTCG-3’ and 5’-ATGGTCGGACATCGAGGAACTCTTGGAAAAGGCGGTGCAGAGCGTCATGAAGGA CGCCGAGTCAATGCAGATGACCTGACTGAGGTTCTTATGGCTCTTG-3’. This cassette was then removed using primers 5’-GGGAAGGCGTGTGCTGCTATACAACTGTACAACGGACGCGCTCGCTGTTTCGGTC TTACGTAGAATCAAGACCTAGGAGCGGGTTAGGGATTGGCTTACC-3’ and 5’-AAAGGCGGTGCAGAGCGTCATGAAGGACGCCGAGTCAATGCAGATGACCAGCG CTGGTAAGCCAATCCCTAACCCGCTCCTAGGTCTTGATTCTACGTAA-3’. These primers result in the insertion of an in-frame V5 tag (underlined) at the extreme C-terminus of *UL135*, linked to *UL135* by a Ser-Ala linker. Virus was recovered by electroporating BACs into HFFF using an Amaxa Nucleofector (program T16). In functional assays virus with either native *UL135,* or *UL135* containing a C-terminal V5 tag behaved similarly, however in the results presented, virus containing a native *UL135* was used in functional assays, and virus containing a C-terminal V5 tag was used in western blot and immunofluorescence assays.

High throughput DNA sequencing

Sequence data sets were generated using either a Genome Analyzer IIx (Sir Henry Wellcome Functional Genomics Facility, University of Glasgow) or MiSeq instrument (in house; Illumina, San Diego, CA, USA). 1000ng of DNA from each sample was sheared by sonication and DNA fragments were purified and size-selected using AMPure XP beads (Beckman-Coulter, High Wycombe, UK). Purified DNA fragments were end-repaired, 3'-adenylated, ligated to paired-end multiplexing adapters and amplified by PCR using standard methods (Illumina). Sequence read data sets were filtered to remove bases less than phred quality 30 and processed for adapter removal using Trim Galore v. 0.2.2, which is a wrapper for Cutadapt and FastQC (http://www.bioinformatics.babraham.ac.uk/projects/trim_galore). Filtered reads were aligned against reference genomes using BWA v0.6.2-r126 (Li and Durbin, 2010). Indexed Bam files were generated from the alignments using Samtools v. 0.1.18 (Li et al., 2009) and viewed using Tablet 1.12.12.05 (Milne et al., 2010).

Generation of recombinant adenoviruses

Recombinant adenoviruses (RAd) were generated using the AdZ system (Stanton et al., 2008). Infection efficiency of RAds on all cell types was initially determined using a RAd expressing eGFP, and subsequently by immunofluorescence for the gene of interest. The *UL135* gene was amplified from Merlin DNA, with a C-terminal V5 tag, using primers 5'-AACCGTCAGATCGC CTGGAGACGCCATCCACGCTGTTTGACCTCCATAGAAGACACCGGGACCGATCC AGCCTGGATCCGCAGGAGAGAAGATCATGGT-3' and 5'-ATAGAGTATACAATAGTGACG TGGGATCCTTACGTAGAATCAAGACCTAGGAGCGGGTTAGGGATTGGCTTACCA GCGCTGGTCATCTGCATTGACTCGG-3', or without any tag. Viruses are all referred to as RAd-UL135. NK assays from Figs. 1 and 2 were repeated using virus lacking or containing a tag, and no difference was detected. In other figures, virus with a V5 tag was used.

Deleting the domains of UL135 that are required for interacting with ABI1/2 and talin was performed by recombineering as previously described (Stanton et al., 2010). For deletion of aa275-308 the amp^r/sacB/lacZ cassette was amplified and inserted using primers 5'-TCGGCCAGCGTCGACACGGTACTGCATCAGCCGCCGCCATCCTGGAAGCCACCTC CGCCGCCCGGGCGCAAGAAGCGGCCTGTGACGGAAGATCACTTCG-3' and 5'-

TAGAGTATACAATAGTGACGTGGGATCCTTACGTAGAATCAAGACCTAGGAGCGGGTTAGGGATTGGCTTACCAGCGCTCTGAGGTTCTTATGGCTCTTG-3’, then removed using primer 5’-TATAGAGTATACAATAGTGACGTGGGATCCTTACGTAGAATCAAGACCTAGGAGCGGGTTAGGGATTGGCTTACCAGCGCTCGACACGATAGGCAGCGTG-3’. For deletion of aa169-205 the amp^r/sacB/lacZ cassette was amplified and inserted using primers 5’-TCGGCCAGCGTCGACACGGTACTGCATCAGCCGCCGCCATCCTGGAAGCCACCTCCGCCGCCCGGGCGCAAGAAGCGGCCTGTGACGGAAGATCACTTCG-3’ and 5’-TACGCGGCGCGGGTATCGGCGTCGGGGGCCTGTGCGACGACAGCCGCGTGGTGGGGGCCCGGACCGGCGGCGTAGGCGGCTGAGGTTCTTATGGCTCTTG-3’. Then removed using primer 5’-AGCCGCCGCCATCCTGGAAGCCACCTCCGCCGCCCGGGCGCAAGAAGCGGCCGCCCACGAAACCCAAGCCGGTCGGCTGGACACCGCCGGTGACGCCCAG-3’.

ABI1 was PCR amplified using primers 5’-CGTCAGATCGCCTGGAGACGCCATCCACGCTGTTTTGACCTCCATAGAAGACACCGGGACCGATCCAGCCTGGATCCACCATGGCA GAGCTGCAGATGTT-3’ (untagged construct) or 5’-TGGAGTTCGTGACCGCCGCCGGGA TCACTCTCGGCATGGACGAGCTGTACAAGGGTAGCGCTGGATCAGCAGGGTCCGCGATGGCAGAGCTGCAGATGTT-3’ (N-terminal GFP tag) along with 5’-GTTTATTGAGT AGGATTACAGAGTATAACATAGAGTATAATATAGAGTATACAATAGTGACGTGGGATCCTTAATCAGTATAGTGCATGATTGATTCAAC-3’. ABI-2 was amplified using primers 5’-CGTCAGATCGCCTGGAGACGCCATCCACGCTGTTTTGACCTCCATAGAAGACACCGGGACCGATCCAGCCTGGATCCACCATGTCCTGCAGATGCTGGAT-3’ (untagged construct) or 5’-TGGAGTTCGTGACCGCCGCCGGGATCACTCTCGGCATGGACGAGCTG TACAAGGGTAGCGCTGGATCAGCAGGGTCCGCGATGTCCTGCAGATGCTGGAT-3’ (N-terminal GFP tag) along with 5’-ACGTTTATTGAGTAGGATTACAGAGTATAACATAGAGTATAATATAGAGTATACAATAGTGACGTGGGATCCTTACTCAGAATAATGCATGATAGACTCA-3’. Templates were pEGFP-C1-Abi1 (a kind gift from Giorgio

Scita/Andrea DiSanza) and pEYFP-Abi2 (a kind gift from Ann-Marie Pendergast) (Courtney et al., 2000).

RAd were recovered by transfecting into 293TREx cells using Effectene (Qiagen), and were subsequently propagated and titrated in 293TREx cells. RAd-UL141 has been described (Tomasec et al., 2005). RAd-Ctrl is a control adenovirus without a transgene insert.

Generation of lifeact expressing cell lines

A DNA sequence encoding the F-actin binding lifeact peptide (Riedl et al., 2008) was generated by annealing of complementary primers 5'-CTAGCGGATCCTAGGCCACCATGGGGGTGG CCGACCTGATCAAGAAGTTCGAGAGCATCAGCAAGGAGGAGCCA-3' and 5'-TGGCTCCTCCTTGCTGATGCTCTCGAACTTCTTGATCAGGTCGGCCACCCCCATGG TGGCCTAGGATCCGCTAG-3'. This sequence was C-terminally tagged with fluorescent proteins by inserting into pcDNA3.1 (Invitrogen, Paisley, UK) encoding mCherry or Citrine fluorescent proteins. Flourescent lifeact constructs were subcloned into a mouse Moloney murine leukaemia virus-based retroviral vector. NKL cells and fibroblasts were stably transfected using the Phoenix retroviral transfection system, with transfectants being selected and maintained in 10 μg/ml blasticidin (Invitrogen).

SILAC

Following in-gel reduction and alkylation, proteins were digested using trypsin with the resulting peptides eluted and dried down for LC-MSMS analysis. LC-MSMS was performed on an OrbiTrap XL coupled to a nanoAcquity. Peptides were resolved using a gradient rising from 7 to 35% acetonitrile by 45 min and 85% acetonitrile by 55 min. MS spectra were acquired at 60,000 resolution (fwhm) with MSMS spectra acquired in the LTQ using a top 6 method and 2 microscans. Samples were acquired in duplicate and raw files processed using MaxQuant v.1.1.1.14. Data were searched, using the embedded search engine Andromeda, against a Uniprot database comprising human and HCMV strain Merlin sequences (downloaded 310112). Carbamidomethyl (C) was set as a fixed modification while oxidised (M), deamidation (NQ) and acetylation (protein N-terminus) were allowed as potential variable modifications. Protein and peptide FDR were set at 0.01. Protein quantitation was based on razor and unique peptides with reported proteins requiring a minimum of 1 ratio count, re-

quantitation was enabled. MaxQuant output was processed in Perseus v.1.1.1.13 to generate significance A values.

Yeast 2 hybrid

IN order to generate a bait strain, the UL135 ORF was amplified using primers 5'-GGCCGGATCCGTATGGTGTGGCT GTGGCTC-3' and 5'-GGCCCTGCAGTCAGGTCATCTGCATTGACT-3' and cloned into BamH1/PstI sites of vector pGBKT7. A yeast bait strain was generated by transforming AH109 cells with this vector. Y187 cells pretransformed with a Human Testis MATCHMAKER cDNA Library (Clontech) were used as prey source.

siRNA

siRNA (Qiagen) were as follows: ABI1 (SI02655338), ABI2 (SI00299075), WAVE2 (SI02664312), talin-1 (SI00086982), talin-2 (SI00109263), CYFIP1 (SI04203717) and Allstars negative control (1027417).

Immunohistochemistry

Primary antibodies were mouse anti-V5 (MCA1360GA, Serotec), anti-ABI1 (a gift from Giorgio Scita (Innocenti et al., 2004)), rabbit anti-V5 (ab9116, Abcam), anti-giantin (ab24586, Abcam), anti-actin (Sigma), anti-WAVE2 (a gift from Dan Billadeau (Nolz et al., 2006)), goat anti-ABI2 (sc-20327, Santa-cruz), anti-NAP1 (N3788, Sigma), anti-talin-1 (TA205, Sigma), anti-talin-2 (121A, abcam), anti-PIR121 (P0092, Sigma) and anti-Vimentin (V6630, Sigma). Secondary antibodies were anti-goat-HRP (Santa-cruz), anti-mouse-HRP and anti-rabbit-HRP (GE Healthcare) or Alexa Fluor 594 and 488 conjugated antibodies (Invitrogen).

Directionality analysis

2 colour Z-stack imaging of NKL/HFFF interfaces was performed to image the actin cytoskeleton either side of the immune synapse. Z-images were collapsed by maximum projection. Directionality of actin fibres in the target cell and the F-actin in at the immune synapse of the NKL cell was determined separately in Fiji/ImageJ using local gradient analysis. The distribution of orientations was fitted to a Gaussian function, the centre of which reports the preferred orientation of the actin cytoskeleton in the respective images.

Supplemental References

Courtney, K.D., Grove, M., Vandongen, H., Vandongen, A., LaMantia, A.S., and Pendergast, A.M. (2000). Localization and phosphorylation of Abl-interactor proteins, Abi-1 and Abi-2, in the developing nervous system. Mol Cell Neurosci *16*, 244-257.

Li, H., and Durbin, R. (2010). Fast and accurate long-read alignment with Burrows-Wheeler transform. Bioinformatics *26*, 589-595.

Li, H., Handsaker, B., Wysoker, A., Fennell, T., Ruan, J., Homer, N., Marth, G., Abecasis, G., Durbin, R., and Genome Project Data Processing, S. (2009). The Sequence Alignment/Map format and SAMtools. Bioinformatics *25*, 2078-2079.

Milne, I., Bayer, M., Cardle, L., Shaw, P., Stephen, G., Wright, F., and Marshall, D. (2010). Tablet--next generation sequence assembly visualization. Bioinformatics *26*, 401-402.

Prod'homme, V., Sugrue, D.M., Stanton, R.J., Nomoto, A., Davies, J., Rickards, C.R., Cochrane, D., Moore, M., Wilkinson, G.W., and Tomasec, P. (2010). Human cytomegalovirus UL141 promotes efficient downregulation of the natural killer cell activating ligand CD112. J Gen Virol *91*, 2034-2039.

Riedl, J., Crevenna, A.H., Kessenbrock, K., Yu, J.H., Neukirchen, D., Bista, M., Bradke, F., Jenne, D., Holak, T.A., Werb, Z., *et al.* (2008). Lifeact: a versatile marker to visualize F-actin. Nat Methods *5*, 605-607.

Stanton, R.J., Baluchova, K., Dargan, D.J., Cunningham, C., Sheehy, O., Seirafian, S., McSharry, B.P., Neale, M.L., Davies, J.A., Tomasec, P., *et al.* (2010). Reconstruction of the complete human cytomegalovirus genome in a BAC reveals RL13 to be a potent inhibitor of replication. J Clin Invest *120*, 3191-3208.

Stanton, R.J., McSharry, B.P., Armstrong, M., Tomasec, P., and Wilkinson, G.W. (2008). Re-engineering adenovirus vector systems to enable high-throughput analyses of gene function. BioTechniques *45*, 659-662, 664-658.

Tomasec, P., Wang, E.C., Davison, A.J., Vojtesek, B., Armstrong, M., Griffin, C., McSharry, B.P., Morris, R.J., Llewellyn-Lacey, S., Rickards, C., *et al.* (2005). Downregulation of natural killer cell-activating ligand CD155 by human cytomegalovirus UL141. Nat Immunol *6*, 181-188.
