# Supplementary material for: HCMV pUL135 Remodels the Actin Cytoskeleton to Impair Immune Recognition of Infected Cells
Source: Cell Host Microbe. 2014 Aug 13;16(2):201–14. doi: 10.1016/j.chom.2014.07.005 (PMC4150922; doi:10.1016/j.chom.2014.07.005)
Supplement: Document S2. Article plus Supplemental Information [file mmc2.pdf]

Cell Host & Microbe
Article

HCMV pUL135 Remodels the Actin Cytoskeleton to Impair Immune Recognition of Infected Cells

Richard J. Stanton,[1,9,*] Virginie Prod'homme,[1,9] Marco A. Purbhoo,[2] Melanie Moore,[1] Rebecca J. Aicheler,[1] Marcus Heinzmann,[3] Susanne M. Bailer,[3,4] Jürgen Haas,[3] Robin Antrobus,[5] Michael P. Weekes,[5] Paul J. Lehner,[5] Borivoj Vojtesek,[6] Kelly L. Miners,[1] Stephen Man,[7] Gavin S. Wilkie,[8] Andrew J. Davison,[8] Eddie C.Y. Wang,[1] Peter Tomasec,[1] and Gavin W.G. Wilkinson[1,*]

[1]Institute of Infection & Immunity, Cardiff University School of Medicine, Heath Park, Cardiff CF14 4XN, UK
[2]Section of Hepatology, Department of Medicine, Imperial College London, London, W2 1PG, UK
[3]Ludwig-Maximilians-Universität München, Max von Pettenkofer-Institut, Pettenkoferstrasse 9a, 80336 München, Germany
[4]Biological Interfacial Engineering, University of Stuttgart, Nobelstrasse 12, 70569 Stuttgart, Germany
[5]University of Cambridge, Cambridge Institute for Medical Research, Addenbrooke's Hospital, Hills Road, Cambridge, CB2 0XY, UK
[6]Regional Centre for Applied Molecular Oncology, Masaryk Memorial Cancer Institute, 65653 Brno, Czech Republic
[7]Institute of Cancer and Genetics, Cardiff University School of Medicine, Heath Park, Cardiff CF14 4XN, UK
[8]Medical Research Council, University of Glasgow Centre for Virus Research, Glasgow G11 5JR, UK
[9]Co-first author
*Correspondence: stantonrj@cf.ac.uk (R.J.S.), wilkinsongw1@cf.ac.uk (G.W.G.W.)
http://dx.doi.org/10.1016/j.chom.2014.07.005
This is an open access article under the CC BY license (http://creativecommons.org/licenses/by/3.0/).

SUMMARY

Immune evasion genes help human cytomegalovirus (HCMV) establish lifelong persistence. Without immune pressure, laboratory-adapted HCMV strains have undergone genetic alterations. Among these, the deletion of the U_L/*b'* domain is associated with loss of virulence. In a screen of U_L/*b'*, we identified pUL135 as a protein responsible for the characteristic cytopathic effect of clinical HCMV strains that also protected from natural killer (NK) and T cell attack. pUL135 interacted directly with abl interactor 1 (ABI1) and ABI2 to recruit the WAVE2 regulatory complex to the plasma membrane, remodel the actin cytoskeleton and dramatically reduce the efficiency of immune synapse (IS) formation. An intimate association between F-actin filaments in target cells and the IS was dispelled by pUL135 expression. Thus, F-actin in target cells plays a critical role in synaptogenesis, and this can be exploited by pathogens to protect against cytotoxic immune effector cells. An independent interaction between pUL135 and talin disrupted cell contacts with the extracellular matrix.

INTRODUCTION

Human cytomegalovirus (HCMV) is a clinically important pathogen, given that it is the leading infectious cause of congenital disorders and frequently associated with severe morbidity and mortality in immunocompromised individuals. HCMV exploits an impressive arsenal of immune evasion genes in order to establish lifelong persistence in its host; the virus is a paradigm of viral immune evasion. The suppression of MHC-I expression by at least four HCMV genes (*US2*, *US3*, *US6*, and *US11*) is well-characterized and promotes evasion of cytotoxic T lymphocytes. However, natural killer (NK) cells play a critical role in controlling CMV infection. Given that MHC-I also constitute the chief NK cell inhibitory ligands, their downregulation has the potential to render infected cells vulnerable to NK cell attack. Our understanding of human NK cell biology and HCMV pathogenesis has been greatly enhanced by studies into how HCMV systematically evades NK cell activation.

To counter NK cells, HCMV encodes a MHC-I homolog (gpUL18), and a peptide derived from the signal peptide of UL40 (SP^{UL40}) stabilizes both gpUL18 and the nonclassical MHC-I molecule HLA-E (Prod'homme et al., 2012; Tomasec et al., 2000). gpUL18 binds the inhibitory receptor LIR-1 1,000-fold more efficiently than endogenous MHC-I (Chapman et al., 1999; Prod'homme et al., 2007), whereas the rescue of HLA-E by SP^{UL40} provides an inhibitory signal via CD94/NKG2A$^+$. NK cells also respond to activating signals. The activating receptor NKG2D binds eight stress proteins (MICA, MICB, and ULBP1–ILBP6), and HCMV encodes at least five genes (*US18, US20, UL16, UL142*, and miR-UL112) that suppress their cell-surface expression (Fielding et al., 2014; Wilkinson et al., 2008). CD112 and CD155 become exposed when infection disrupts intercellular contacts and serve as ligands for the activating receptors CD226 and CD96, and gpUL141 suppresses cell-surface expression of both (Prod'homme et al., 2010; Tomasec et al., 2005). Moreover, the tegument protein pp65 binds directly to NKp30, inhibiting NK cell cytotoxicity (Arnon et al., 2005). NK cells can also kill target cells through direct ligation of death receptors, and gpUL141 contributes toward NK cell evasion by downregulating TRAIL receptor 2 (Smith et al., 2013).

HCMV laboratory-adapted strains have accumulated genetic defects during in vitro passage (Dargan et al., 2010; Stanton et al., 2010), the most extreme being a 15 kb deletion of the U_L/*b*' region (genes *UL133–UL150*) in strain AD169. Loss of U_L/*b*' correlates with greatly enhanced sensitivity to NK cell attack (Cerboni et al., 2000; Tomasec et al., 2005; Wang et al., 2002), which is ascribed in part to loss of *UL141* and *UL142*. In

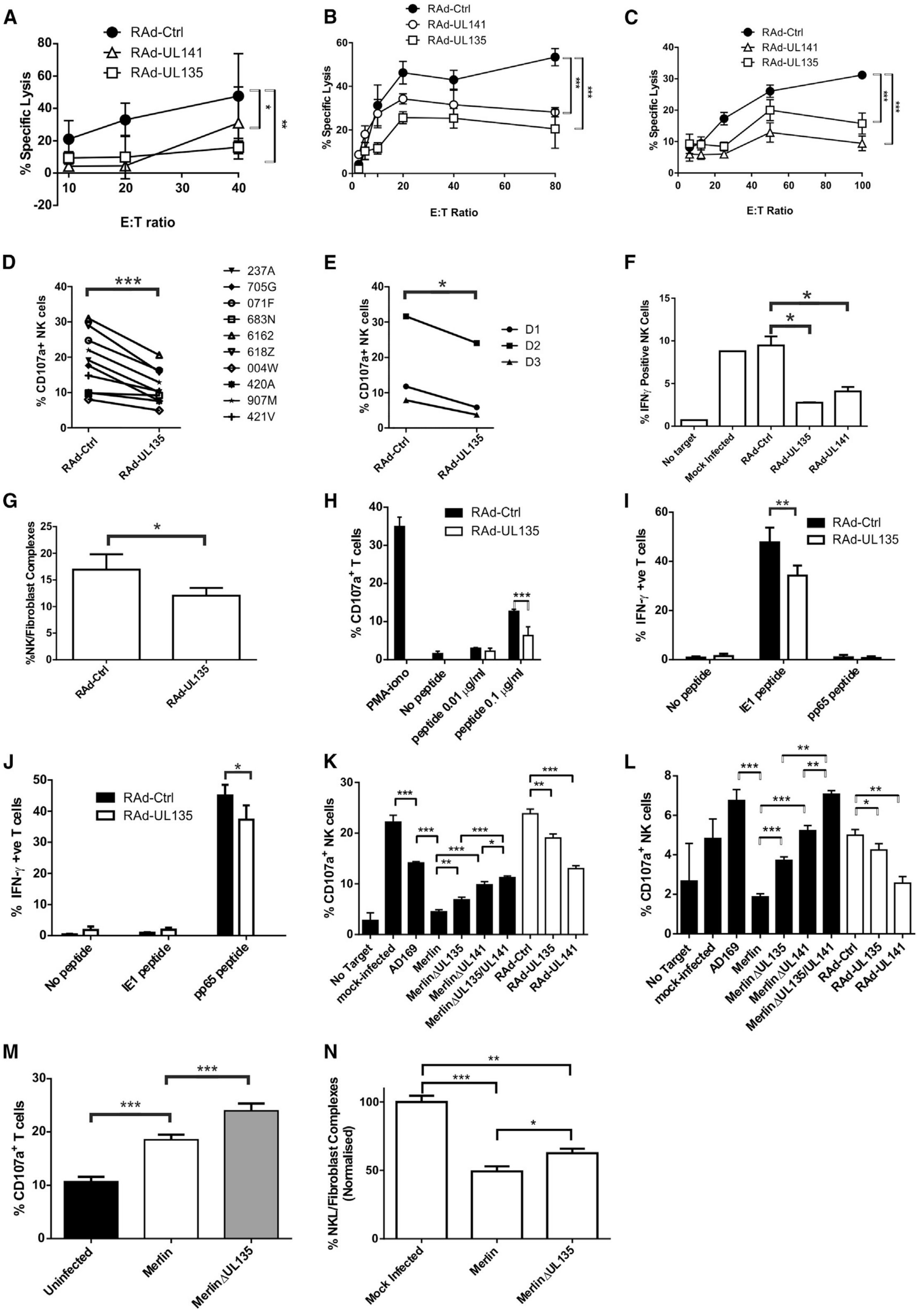

(legend on next page)

Table 1. NK Clone Cytotoxicity

D3 NK Clones	Activation[a]	Inhibition[b]	No Change[c]	No Killing[d]
RAd-UL141	4/28 (14.3%)	7/28 (25%)	10/28 (35.7%)	7/28 (25%)
RAd-UL135	0/28 (0%)	14/28 (50%)	5/28 (17.9%)	9/28 (32.1%)
**D2 NK Clones**	**Activation**	**Inhibition**	**No Change**	**No Killing**
RAd-UL141	3/29 (10.3%)	12/29 (41.4%)	9/29 (31%)	5/29 (17.2%)
RAd-UL135	0/29 (0%)	18/29 (62.1%)	6/29 (20.7%)	5/29 (17.2%)
**All NK Clones**	**Activation**	**Inhibition**	**No Change**	**No Killing**
RAd-UL141	7/57 (12.3%)	19/57 (33.3%)	19/57 (33.3%)	12/57 (21%)
RAd-UL135	0/57 (0%)	32/57 (56.1%)	11/57 (19.3%)	14/57 (24.6%)

Data show the number and percentage of NK clones isolated from PBMC.
[a]Activated NK clones kill RAd-UL141/RAd-UL135-infected autologous SF with specific lysis > 10% in comparison to RAd-Ctrl.
[b]Inhibited NK clones kill RAd-Ctrl-infected SF with specific lysis > 10% in comparison to RAd-UL141 or RAd-UL135.
[c]NK clones with no change kill RAd-UL141 or RAd-UL135-infected SF with specific lysis < 10% in comparison to RAd-Ctrl.
[d]NK clones with no killing kill RAd-Ctrl-infected SF with specific lysis < 10%.

a systematic screen, we identified *UL135* as a third NK cell evasion function within U_L/b'. pUL135 was unique in providing efficient protection against not only an exceptionally broad range of NK as well as T cells. Classically, NK cell modulatory functions either promote cell surface expression of inhibitory ligands or suppress activating ligands; however, pUL135's mechanism of action is different. Dynamic remodeling of the actin cytoskeleton within effector cells plays a major role in organizing the immune synapse (IS) and promoting degranulation in cytotoxic T lymphocytes (CTLs) and NK cells. Biochemical, genetic, and functional studies performed on pUL135 identified an interaction with abl interactor 1(ABI1) and ABI2, recruitment of the WAVE2 complex, and subsequent actin depolymerization in target cells as critical in evading NK cell recognition. Direct imaging identified an association between actin in the target cell and the IS in the effector cell, and *UL135* expression results in a reduced capacity to organize the IS in the effector cell. This HCMV NK evasion function implies that the actin cytoskeleton in target cells plays a key role in IS formation and proposes another mechanism by which infectious agents can evade immune recognition.

RESULTS

UL135 Inhibits NK Cells

Given that the HCMV U_L/b' region plays a major role in promoting resistance to NK cell attack, 19 genes encoded within U_L/b' region were inserted into a replication-deficient adenovirus (RAd) vector, and their expression was validated (Figure S1A available online). Then, this expression library was systematically screened in NK-cell-functional assays, and pUL135, gpUL141, and gpUL142 were identified as eliciting significant protection against NK-cell-mediated cytolysis (Figure S1B). As recognized NK cell modulatory functions, gpUL141 and gpUL142 served as positive controls, whereas the protection elicited by pUL135 provided evidence that this gene had an NK cell modulatory function.

The levels of protection elicited by pUL135 rivalled those achieved with gpUL141. In a representative chromium release assay, pUL135 reduced specific lysis by 64% at an E:T ratio of 40:1 with the immortalized NKL cell line (Figure 1A). In cytolysis assays, pUL135 also elicited protection against a heterogeneous primary NK cell population, irrespective of whether assays were performed in an allogeneic (Figure 1B) or an autologous setting (Figure 1C). In the autologous assay, pUL135 elicited 49.5% inhibition of NK-cell-mediated cytolysis at an E:T ratio of 100:1. Moreover, using a CD107-mobilization assay, protection was elicited against IFN-α-activated NK cells from 13 different donors. In allogeneic assays, NK cell degranulation of ten bulk cultures was significantly inhibited by pUL135 expression with a mean difference of 36.8% inhibition (Figure 1D). Efficient pUL135-mediated inhibition was also observed in autologous NK degranulation assays with peripheral blood mononuclear cell (PBMC) bulk cultures from three other donors with an average inhibition of 42.2% (Figure 1E). A panel of 57 NK cell clones from two donors were expanded following single-cell sorting (Table 1). In autologous assays, 25 of 57 clones were either unable to kill target cells or not substantially affected by

Figure 1. UL135 Decreases NK Cell Cytotoxicity, Degranulation, and IFN-γ Production and Adhesion as Well as T Cell Degranulation and IFN-γ Production

(A–E) Cytotoxicity assays were set up with the NKL cell line against HFFF-hCAR (A), IFN-α-activated T-cell-depleted PBMC against HFFF-hCAR (B), or IFN-α-activated T-cell-depleted PBMC against autologous skin fibroblasts (SFs; C). NK cell degranulation assays were performed with IFN-α-activated PBMC against HFFF-hCAR (D) or autologous SF (E).
(F) Intracellular IFN-γ stainings were set up with IFN-α-activated PBMC against HFFF-hCAR.
(G) Adhesion assays were performed with HFFF-hCAR infected with RAd-UL135 or empty control vector (RAd-Ctrl) and NK cells.
(H) T cell degranulation assays were set up with the 7E7 T cell clone against MRC-5 pulsed with antigenic peptide. T cells incubated with PMA-iono or without peptide are shown as controls.
(I–J) T cell intracellular IFN-γ staining assays were set up with the D7IE1 (I) and D7pp65 (J) T cell lines against autologous SF infected with RAd-Ctrl or RAd-UL135 and pulsed with peptide.
(K–L) NK cell degranulation assays were set up with IFN-α-activated PBMC against allogeneic HFFF-hCAR (K) or autologous SF infected for 48 hr with HCMV strains AD169, Merlin, MerlinΔUL135, MerlinΔUL141, MerlinΔUL135/ΔUL141, or RAd-UL135, RAd-UL141, and RAd-Ctrl (L).
(M) T cell degranulation assays were set up with a pp65-specific T cell line against autologous SF either mock infected or infected with Merlin or MerlinΔUL135.
(N) Adhesion assays were performed with HFFF infected with the indicated viruses and NK cells. Results from three experiments were normalized and combined.
Results are means ± SD of triplicate (A–C, G, H, and N), duplicate (D–F), or quadruplicate (I–M) samples and are representative of three independent experiments (A–M). Two-way ANOVA test. *p < 0.05, **p < 0.01, ***p < 0.001.

the expression of pUL135. The remaining NK cell clones (56.1%) were all inhibited by pUL135. Although 33% of clones were inhibited by gpUL141, 12.3% were still activated. In contrast, pUL135 was exclusively associated with an inhibitory response in this series of experiments. In addition to direct killing of target cells by cell-mediated cytotoxicity, inflammatory cytokines released by NK cells play a crucial role in suppressing virus replication; IFN-γ expression was suppressed when NK cells were cocultured with targets expressing either pUL135 or gpUL141 in comparison to vector control (Figure 1F). NK activation requires the establishment of cell:cell contact culminating in formation of an IS, and UL135 reduced the efficiency of NK cells adhesion to fibroblast targets (Figure 1G).

When expressed in isolation, pUL135 inhibited the NKL cell line, primary NK cell cultures from >13 different donors, and the majority of NK cell clones tested. Protection was observed in both an allogeneic and autologous setting. Moreover, pUL135 suppressed NK-cell-mediated cytolysis, degranulation, IFN-γ expression, and adhesion to target cells.

UL135 Inhibits CD8$^+$ T Cells

Next, we expanded the breadth of the study by investigating whether pUL135 impacted T cell recognition. A HLA-A*0201-restricted T cell clone specific for a human papillomavirus 16 E6 epitope was coincubated with MRC-5 fibroblasts (HLA-A*0201) loaded with specific antigenic peptide (Figure 1H). The proportion of CD107a$^+$ T cells increased with the amount of specific peptide loaded on target cells, and pUL135-specific inhibition occurred in the presence of 0.1μg/ml peptide. To further characterize this effect, IFN-γ production was measured from two HCMV-specific HLA-A*0201-restricted T cell lines generated from a donor with tetramer detectable responses against dominant IE1 and pp65 epitopes. At assay, the T cell lines contained >70% tetramer-positive cells. When incubated with autologous fibroblasts coated with specific peptide, there was a strong pUL135-specific inhibition of IFN-γ production (Figures 1I and 1J). Therefore, pUL135 efficiently suppressed both NK cell and T cell functions.

UL135 Functions during Productive HCMV Infection

An important test for any HCMV immune evasion gene is whether it functions in the context of virus infection. The HCMV strain AD169 lacks the U_L/b' sequence, and therefore *UL135*, *UL141*, and *UL142*, and provides substantially less protection against NK cells than strain Merlin. Deletion of either *UL135* or *UL141* from strain Merlin resulted in an increase in NK cell degranulation, whereas deletion of both genes had an additive effect. Comparable results were obtained when primary NK cells from volunteer donors were tested against allogeneic (Figure 1K) or autologous cells (Figure 1L). The ability of pUL135 to protect against T cell degranulation was also tested in the context of HCMV infection (Figure 1M). A particularly active pp65-specific T cell line recognized autologous HCMV-infected cells despite viral-mediated downregulation of MHC-I, and deletion of *UL135* from the HCMV genome resulted in an increase in T cell degranulation. Merlin reduced the ability of infected cells to form conjugates with NK cells, and this ability was inhibited when *UL135* was deleted from the genome (Figure 1N). Whether expressed in isolation or during productive HCMV infection, pUL135 promoted evasion of both NK and T cells.

UL135 Expression

UL135 exhibits a high degree of sequence conservation in characterized HCMV strains and clinical isolates. An ortholog is present in chimpanzee cytomegalovirus (CMV) but not CMV species of the lower primates (Umashankar et al., 2011). pUL135 is exceptionally proline-rich (60 of 308 amino acids [aa]), contributing to predictions that it contains 22 potential SH3 binding sites and is 80% "structurally disordered." pUL135 was expressed at slightly higher levels from RAd-UL135 in comparison to HCMV (Figure S2A) and was synthesized as two species with molecular masses of 38kDa and 40kDa in comparison to a predicted size of 33 kDa. During productive HCMV infection, pUL135 was expressed during early phase (24 hr), but, unusually for an HCMV gene, levels declined through the late phase (Figure 2A). pUL135 is membrane associated (Umashankar et al., 2011) and predicted to contain an N-terminal transmembrane domain and two *N*- and 42 *O*-linked glycosylation sites. However, it was not sensitive to PNGaseF or O-glycosidase, indicating these glycosylation sites were not used (Figure S2B). In order to optimize detection in the context of HCMV infection, a sequence providing a C-terminal V5 tag was inserted into *UL135*. When expressed in isolation from RAd-UL135,or in the context of HCMV infection, pUL135 colocalized with markers of the Golgi apparatus and at the plasma membrane (Figure 2B). Golgi localization was more prominent when UL135 was expressed in isolation, most likely because of the expression of other HCMV genes and reorganization of the Golgi apparatus, during infection. A monoclonal antibody raised to pUL135 was able to detect the protein on cells only once they had been permeabilized (Figure 2C). The data imply that pUL135 is anchored in the Golgi and plasma membranes via its N-terminal hydrophobic domain with the main body of the protein exposed to the cytosol, which is consistent with previous data (Umashankar et al., 2011). This topological orientation in the plasma membrane would make it difficult for pUL135 to act directly as an inhibitory ligand.

UL135 Remodels the Actin Cytoskeleton

Expression of pUL135 in fibroblasts consistently induced dramatic changes in cell morphology, and focal adhesions and cell projections are lost as cells are rounded up (Figures 2D and S2C). Even in the context of productive infection, pUL135 clearly influenced the HCMV-induced cytopathic effect. In contrast to parental virus, cells infected with MerlinΔUL135 were clearly "flatter" and more spread out, which is indicative of enhanced adherence (Figures 2E and S2D). The actin cytoskeleton was stained with phalloidin; pUL135 expression resulted in elimination of F-actin stress fibers from the cell center, whereas the cortical actin matrix underlying the plasma membrane appeared reinforced and colocalized with pUL135 (Figures 3A and S3A). During productive HCMV infection, F-actin was lost from the center of infected cells, and UL135 colocalized with cortical actin. However, in the absence of pUL135, a substantial proportion of F-actin remained in the center of infected cells (Figures 3B and S3B). In contrast, microtubules and intermediate filaments were not overtly displaced by pUL135 (Figures S3C–S3F).

An Intact Actin Cytoskeleton Is Required for NK Cell Recognition

pUL135 remodeled the actin cytoskeleton and inhibited both NK and T cell activation. NK and T cell modulatory functions generally act by either promoting cell-surface expression of an inhibitory ligand or suppressing an activating ligand; however, pUL135 did not alter the expression of any candidate ligands or adhesion molecules (Table S1). Therefore, we investigated whether pUL135's capacity to remodel the cytoskeleton was involved in immune evasion. The inhibitor of actin polymerization latrunculin A (LatA) reproducibly suppressed NK cell activation against control cells in a concentration-dependent manner, ultimately reaching levels comparable to those achieved with pUL135 (Figure 3C). Comparable results were obtained following HCMV infection in the presence or absence of pUL135 (Figure 3D). Remarkably, in the presence of pUL135, LatA did not suppress NK cell activation. The fact that the effects of LatA and pUL135 were not additive suggested that they exerted their effect via the same mechanism. In support of this hypothesis, F-actin in cells expressing pUL135 (Rad-UL135/Merlin) was unaffected by LatA treatment. In contrast, cells lacking pUL135 (RAd-Ctrl/MerlinΔUL135/Mock) displayed progressive loss of central actin as LatA concentrations increased until, at high concentrations, they resembled pUL135-expressing cells; i.e., a rounded cell devoid of central actin fibers (Figures 3E, S3G, and S3H).

Similar results were obtained with the actin-stabilizing drug jasplakinolide. Treatment of fibroblasts with jasplakinolide led to loss of stress fibers and the formation of an F-actin aggresome (Figure S3I), which correlated with a reduction in NK degranulation (Figure S3J). Treatment of cells expressing pUL135 with moderate concentrations of jasplakinolide had little effect on pUL135-mediated actin remodeling or NK degranulation. At high concentrations, there was complete loss of stress fibers in both control and pUL135-expressing cells, and NK degranulation was equivalent in both.

Thus, actin is required for NK recognition of targets, and, following modification of actin by LatA or jasplakinolide, control cells inhibit NK degranulation to the same extent as pUL135-expressing cells.

UL135 Interacts with Members of the WAVE2 Regulatory Complex and Talin

Yeast two-hybrid assays and stable isotope labeling by amino acids in cell culture (SILAC) immunoprecipitation experiments were undertaken in order to identify pUL135-interacting partners. In the yeast two-hybrid screen, ABI1 and ABI2 were both identified in multiple clones. This finding was consolidated and expanded when SILAC immunoprecipitation experiments performed on pUL135-expressing fibroblasts

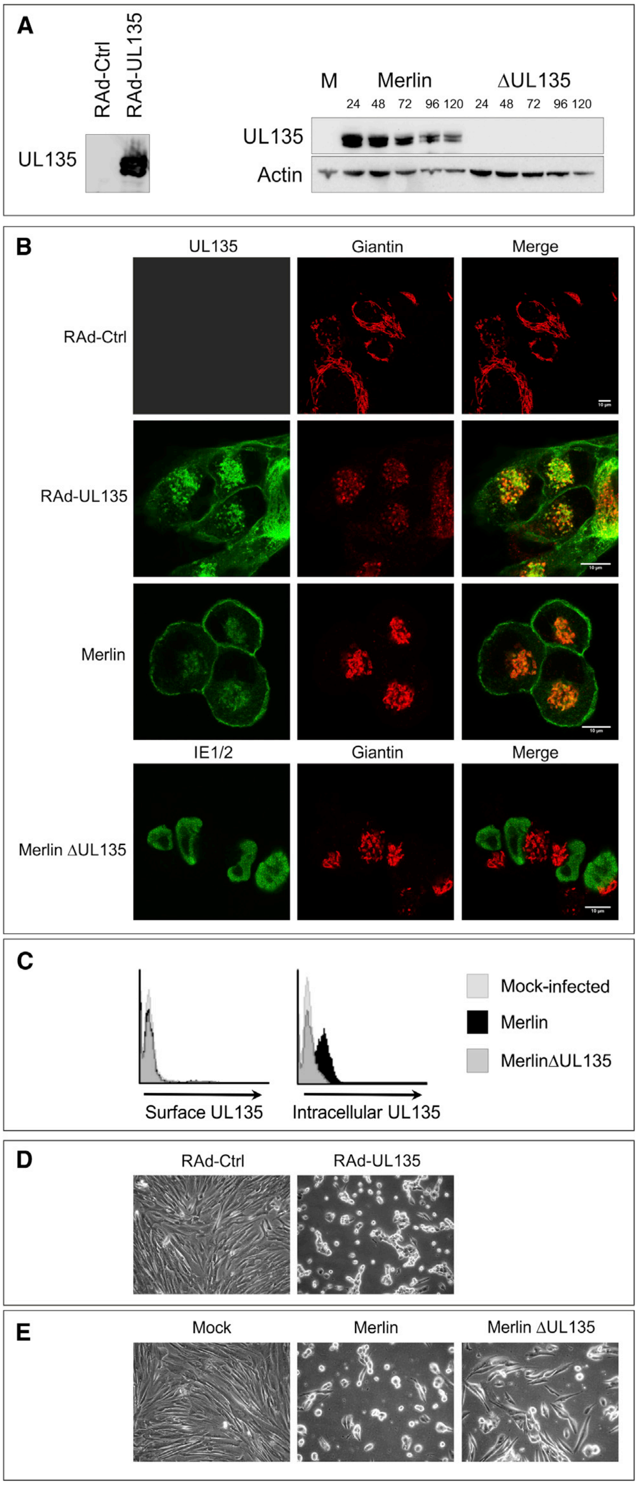

Figure 2. Expression of UL135

(A) HFFF-hCAR were infected with RAd-UL135 or an empty control vector (RAd-Ctrl) for 48 hr, and HFFF were mock infected (M) or infected with HCMV strain Merlin or MerlinΔUL135 for the indicated times. Proteins were separated by SDS-PAGE, and western blot was performed for UL135 (V5 antibody) or actin.

(B) HFFF-hCAR were infected with RAd-UL135 or RAd-Ctrl for 48 hr, and samples were stained for UL135 (V5 antibody) and giantin (golgi marker). HFFF were infected with the indicated strains of HCMV for 48 hr and stained for UL135 (V5 antibody) or IE1/IE2 (MerlinΔUL135) and giantin.

(C) HFFF were infected with the indicated strains of HCMV and stained for either surface or intracellular UL135 expression 48 hr later.

(D and E) Brightfield images of HFFF-hCAR infected with RAd-UL135 or RAd-Ctrl (D) and HFFF (E) mock infected or infected with HCMV strain Merlin or MerlinΔUL135 for 48 hr.

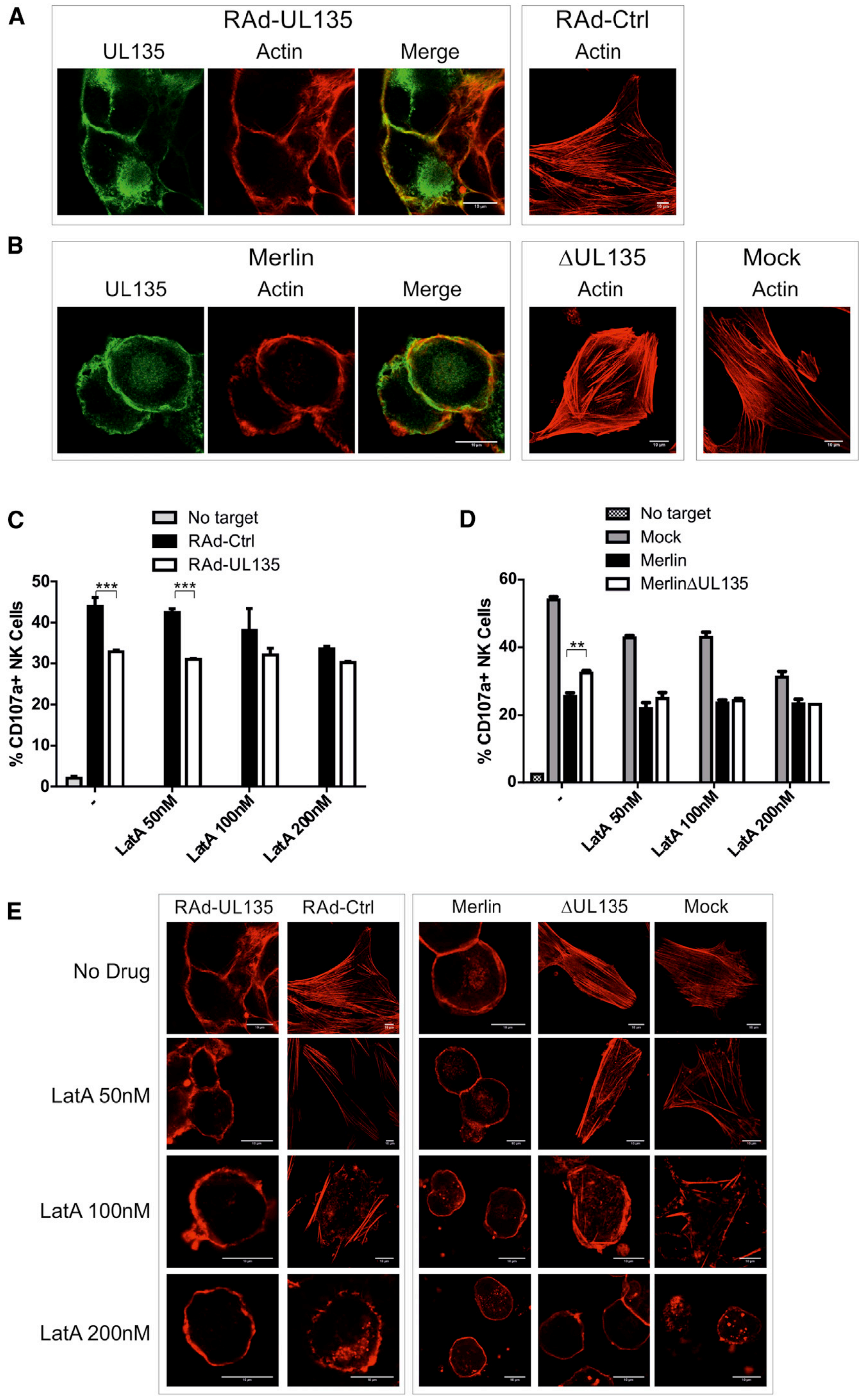

Figure 3. UL135 Modifies Actin Structure

(A–D) HFFF-hCAR were infected with RAd-UL135 or RAd-Ctrl (A and C), or HFFF were infected with HCMV strain Merlin or MerlinΔUL135 or mock infected (B and D). Samples were stained 48 hr postinfection for UL135 (V5 antibody) and actin (phalloidin; A and B). Following infection, samples were incubated with the indicated doses of LatA (LatA; C and D).NK cell degranulation assays were performed 48 hr postinfection with IFN-α-activated PBMC. Results are mean ± SD and are representative of three independent experiments. Two-way ANOVA test with post tests were performed between RAd-Ctrl and RAd-UL135 (C) or Merlin and MerlinΔUL135 (D). *$p < 0.05$, **$p < 0.01$, ***$p < 0.001$.

(E) Samples treated as in (C and D) were stained for actin (phalloidin).

identified WAVE2, ABI1, NAP1, CYFIP1, and talin-1 (Table S2). Significantly ABI1, ABI2, NAP1, CYFIP1, and WAVE2 itself are all components of the WAVE2 regulatory complex (WRC), which regulates the actin nucleator Arp2/3 (Takenawa and Suetsugu, 2007). The interaction with WAVE2, ABI1, ABI2, NAP1, CYFIP1, and talin-1 were validated in conventional immunoprecipitation experiments when pUL135 was expressed in both isolation and the context of HCMV infection (Figure 4A).

ABI1 (Figures 4B and 4C), ABI2 (Figures S4A and S4B), and WAVE2 (Figures 4D and 4E) all exhibited a diffuse cellular distribution that excluded the nucleus as well as a marked association with membrane protrusions, consistent with their role in actin remodelling. Whether pUL135 was expressed in isolation or during productive HCMV infection, all three cellular proteins trafficked with pUL135 to the Golgi apparatus (Figures S4C–S4F) and colocalized evenly around the plasma membrane. ABI1 and ABI2 were more readily imaged in cells expressing pUL135, even though their absolute abundance remained constant as assessed by western blot and flow cytometry (Figures 4F and 4G; data not shown). pUL135 also efficiently colocalized with ABI1 and ABI2 when expressed with a C-terminal GFP tag (Figures S4G and S4H). pUL135 interacted and localized with ABI1, ABI2, and WAVE2 at the Golgi apparatus and plasma membrane without changing their overall abundance.

Talin-1 binds actin and participates in focal adhesions but is not a member of the WRC. pUL135 expression alone was sufficient to recruit talin-1 to the Golgi apparatus and cell membrane, but, rather than stimulating cell adhesion, focal adhesions were disrupted (Figure 4H). pUL135 is not needed for HCMV to disrupt focal adhesions (Stanton et al., 2007) yet was required for talin-1 to be redistributed to the cell periphery during HCMV infection (Figure 4I).

UL135 Inhibits Cytoskeletal Remodelling

pUL135 consistently induced cell rounding, loss of contact adhesions, and disassembly of stress fibers. RNA interference was used to investigate the roles played by the various

pUL135-interacting cellular proteins (Figure S5A). Ablation of both ABI1 and ABI2 (ABI1/ABI2) was associated with a very slight reduction in F-actin levels in control cells, consistent with a requirement for ABI1/ABI2 in the WRC (Figures 5A andS5B). In contrast, pUL135 expression induced a very dramatic loss of F-actin. Moreover, following ABI1/ABI2 knockdown, pUL135 lost the capacity to depolymerize F-actin fibers throughout the center of the cell. This implies that, rather than directly inhibiting ABI1/ABI2, pUL135 is commandeering the WRC to actively promote F-actin depolymerization. To determine whether the entire WRC was involved in pUL135-mediated actin remodelling, the WRC member CYFIP1 was targeted with small interfering RNA (siRNA). Although knockdown had no obvious effect on actin, CYFIP1 was required for pUL135-mediated depolymerization of actin (Figures 5B and S5C). Therefore, ABI1/ABI2 was not the only WRC component necessary for pUL135-mediated actin remodelling.

Talin binds the cytosolic domain of integrins at focal adhesions, at which point it provides a bridge between the actin cytoskeleton and the extracellular matrix (Kim et al., 2011), playing a key role in activating integrin function in inside-out signaling. pUL135 inhibited cell spreading on fibronectin; this phenotype was also inhibited by knockdown of ABI1/ABI2 but not talin-1 and talin-2 (Figures 5C and 5D). In order to investigate the interaction between pUL135 and the WRC, pUL135 was immunoprecipitated from cells in which ABI1/ABI2 had been knocked down (Figures 5E and S5D). Knockdown of any member of the WRC can lead to loss of other members of the complex, yet the interaction of pUL135 with talin was clearly unaffected, which is consistent with pUL135 binding talin independently of its interaction with the WRC.

A series of in-frame deletions were generated through *UL135*, and the resulting gene products tested for their capacity to bind ABI1/ABI2 and talin (Figure S5E). The center of pUL135 (aa 169–206) was required for the interaction with the WRC, but not talin. The C terminus (aa 275–308) was required for the interaction with talin (Figures 5F and S5F). In agreement with siRNA studies, the interaction between pUL135 and the WRC was responsible for the loss of central F-actin fibers (Figures 5G and S5G). The Δ169–206 mutant failed to redistribute ABI1/ABI2, which instead concentrated in membrane protrusions around the periphery of the cell (Figure 5H, arrowed). Likewise, deletion of the talin-binding domain (aa 275–308) resulted in talin no longer colocalizing with pUL135 but instead concentrating in punctate structures around the cell periphery (Figure 5I, arrowed). Deletion of either the ABI1/ABI2 or talin interaction domains partially restored the capacity of cells to spread on a fibronectin matrix, and deletion of both domains eliminated the phenotype completely (Figure 5J). Thus, pUL135 affected cell adhesion by two mechanisms: (1) through the interaction with ABI1/ABI2, pUL135 actively disassembled the actin framework that the extracellular matrix is attached to and, (2) interacting directly with talin, pUL135 disrupted focal adhesions and suppressed integrin function. Targeting ABI1/ABI2 by either siRNA or mutation released the WRC and restored cell spreading. Deleting the talin-interacting domain also restored cell spreading, but talin knockdown in pUL135-expressing cells did not, implying that pUL135 directly suppressed talin function.

The Interaction of UL135 with ABI1/ABI2 Is Required for Inhibition of NK Cells

The functional significance of the interaction of pUL135 with ABI1/ABI2 and talin in eliciting protection against NK cells was investigated with siRNA. The interaction with ABI1/ABI2, rather than talin, was critical for NK cell evasion whether assessed by CD107a mobilization (Figures 6A and 6B) or cytolysis assays (Figures 6C and 6D). This correlation was supported in experiments utilizing the characterized pUL135 deletion mutants. Only versions of pUL135 that bound ABI1/ABI2 elicited protection against NK cells (Figure 6E). Indeed, pUL135 functioned even more effectively as an NK evasion function when the talin-interacting domain was deleted. pUL135's direct interaction with ABI1/ABI2 was not sufficient, a second WRC member (CYFIP1) was also required for pUL135 in order to induce protection against NK cell attack (Figure 6F). The recruitment of the entire WRC is thus implicated in pUL135 function. In the absence of pUL135, neither the ablation of ABI1/ABI2 nor CYFIP1 had an impact on NK cell recognition, indicating that pUL135 used ABI1/ABI2 to recruit the WRC and then subverted its activity to suppress NK cells.

Additional evidence for this hypothesis was provided in experiments in which ABI1/ABI2 was overexpressed. Overexpression of ABI1/ABI2 in the presence of pUL135 resulted in increased recruitment of ABI by UL135, yet it decreased the recruitment of WAVE2 (Figure S6A). This was interpreted as being due to an excess of monomeric ABI1/ABI2, which saturated pUL135 and prevented the recruitment of ABI1/ABI2 found in the WRC. This correlated with reduced modification of actin by pUL135 (Figures S6B and S6C) and a reduced ability for pUL135 to inhibit NK degranulation (Figure 6G). Overexpression of ABI1/ABI2 in the absence of pUL135 did not affect NK degranulation.

UL135 Inhibits the Formation of an Immune Synapse

pUL135-mediated protection against immune effector cells depended on its interaction with ABI1/ABI2 and capacity to disrupt and/or remodel the actin cytoskeleton. Furthermore, a specific inhibitor of actin polymerization recapitulated this effect. Moreover, NK cells were less able to adhere to cells expressing pUL135. To progress our understanding of pUL135 function, the IS was imaged directly during NK cell recognition. Following coculture of fibroblasts with NK cells, bona fide ISs were readily detected at sites of cell:cell contacts, as demonstrated by a ring of polymerized actin indicative of NK cell activation and synapse formation (Figure 6H, RAd-Ctrl). When pUL135 was expressed in target cells, ISs were less common, their morphology was less defined, and actin polymerization at the IS was much reduced (Figures 6H and 6I). Phalloidin selectively labeled long F-actin filaments within the target cell. Interestingly, these frequently delineated the inner or outer limits of actin polymerization within the actin ring formed by the activated NK cell (Figure 6J). To unambiguously differentiate the actin in the target cell from that in the NK cell, fibroblasts were engineered to express a Citrine-labeled live-cell actin marker lifeact, whereas NKL cells expressed mCherry-tagged lifeact. Fluorescent labeling clearly differentiated between the "red" actin ring associated with the IS in the NK cell and the underlying "green" actin fibers within the target cell (Figures 6K–6M). Like phalloidin staining, synapses were much less common when UL135 was expressed in

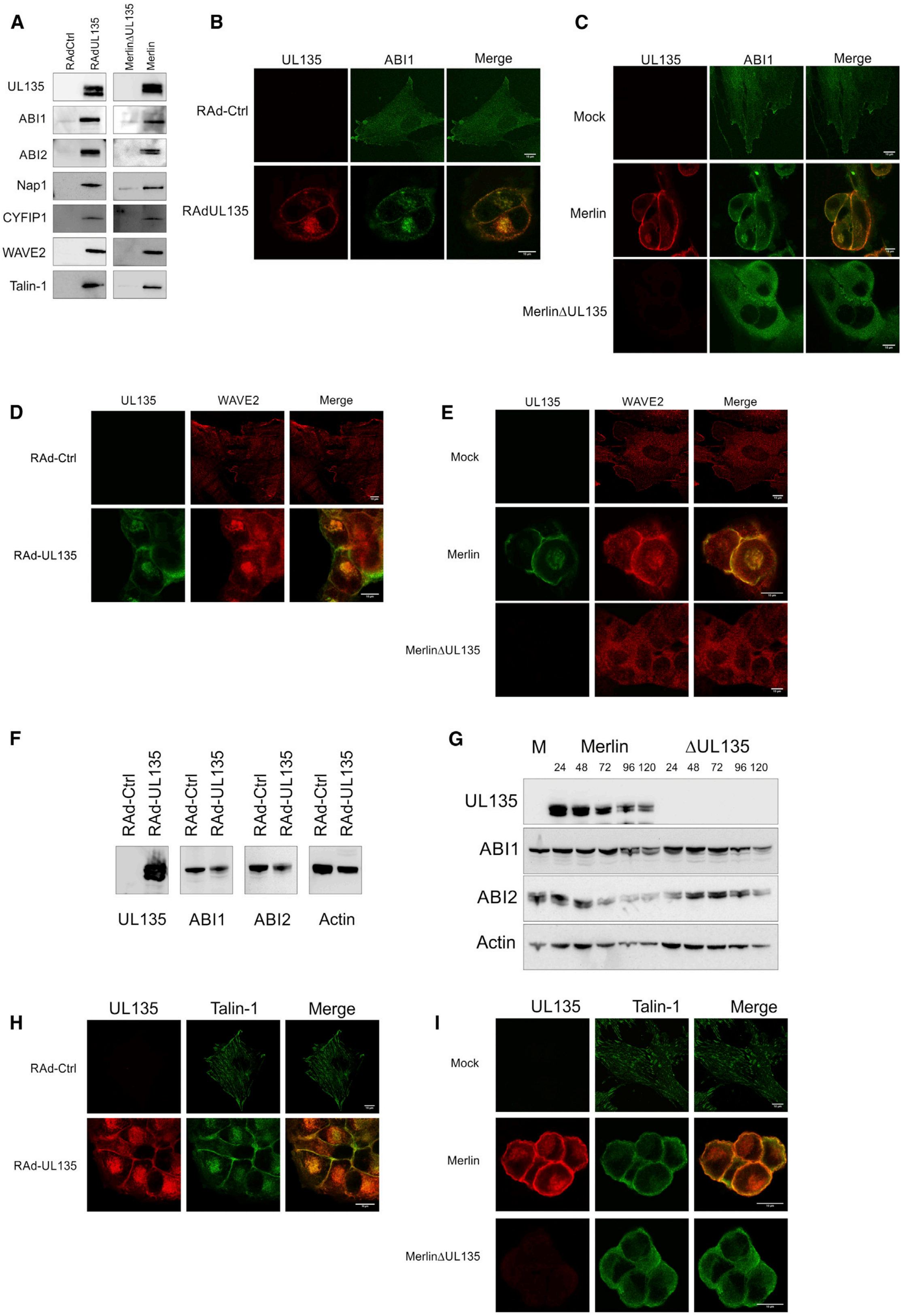

(legend on next page)

the target cell, and, when they did form, the characteristic actin structure was absent (Figure 6K). Lifeact imaging also revealed that the actin ring within the NK cell was delineated by, and commonly aligned with, the actin fibers within the target cell (Figures 6L and S6D). Moreover, we observed that actin polymerization within the actin ring formed by the effector cell was clearly less dense where it "intersected" with actin fibers in the target cell (Figure 6M), consistent with the structure of the cytoskeleton within the target cell influencing synapse formation in the effector cell. Therefore, we propose that F-actin filaments within the target cell play an important role in the formation and structure of the synapse in the NK cell, and pUL135 may inhibit synaptogenesis by derailing this process.

DISCUSSION

The U_L/*b*' sequence (*UL133–UL150*) is an important determinant of HCMV pathogenesis in vivo that harbors a striking concentration of genes implicated in controlling host immunity; these include a potentiator of tumor necrosis factor α (TNF-α) signaling (*UL138*) (Le et al., 2011), NK cell evasion functions *UL141* and *UL142* (Wilkinson et al., 2008), TNF receptor homolog *UL144* (Benedict et al., 1999), and IL-8-like virokine *UL146* (Penfold et al., 1999). By promoting evasion of both NK and T cell recognition, pUL135 can be expected to contribute toward the increased virulence bestowed by HCMV U_L/*b*'. Moreover, being encoded within the latency-associated *UL133–UL138* transcriptional unit, UL135 has the potential to confer immune protection during virus reactivation in differentiating myeloid cells. Nevertheless, pUL135 is efficiently expressed during productive infection, reaching peak levels at 24 hr postinfection, and makes a major contribution toward the characteristic cytopathic effect of clinical HCMV strains.

The profound effect pUL135 exerts on cellular morphology was mediated by two distinct mechanisms acting independently through talin and ABI1/ABI2. Integrins are heterodimeric integral membrane proteins that link the cytoskeleton with extracellular matrix (Kim et al., 2011). Bidirectional signaling through integrins plays a crucial role in regulating cell proliferation, survival, transcription, migration, and cytoskeletal organization. The N-terminal FERM domain of talin attaches to the cytoplasmic tail of the β-integrin subunit, whereas its C-terminal rod domain binds actin. Talin is not merely a molecular bridge but a key regulator of inside-out signaling and, hence, integrin activation. pUL135 and talin-1 participated in a stable complex at the plasma membrane that correlated with disruption of focal adhesins and suppression of contacts with the extracellular matrix. Although integrins play a major role in immune recognition by cytotoxic cells, talin knockdown had no discernible impact on pUL135's capacity to suppress NK cell function. Indeed, deletion of the talin binding domain actually increased the ability of pUL135 to inhibit NK cells. Beyond immune evasion, HCMV controls the motility and differentiation of infected cells in order to promote virus dissemination, and infection of endothelial cells can promote transendothelial migration of infected monocytes by increasing the permeability of the endothelium (Bentz et al., 2006). By inhibiting the ability of the cell to interact with the extracellular matrix, pUL135 has the potential to affect these processes.

The adaptor proteins ABI1 and ABI2 play a major role in promoting actin polymerization through their interaction with mena (Tani et al., 2003), the diaphenous-like formins (Ryu et al., 2009), N-WASP, and WAVE1–WAVE3 (Takenawa and Suetsugu, 2007). We demonstrated that pUL135 bound directly to ABI1/ABI2 and recruited members of the WRC, including WAVE2, CYFIP1, and NAP1. The WRC promotes actin polymerization following recruitment of profilin, actin, and the Arp2/Arp3 complex. However, in recruiting the WRC rather than promoting actin polymerization, pUL135 induced the selective loss of stress fibers, whereas cortical actin was preserved. The effect is most readily observed when pUL135 is expressed in isolation, given that HCMV encodes additional functions that impact on actin (Seo et al., 2011) and adhesion junctions (Stanton et al., 2007).

Remodeling of the actin cytoskeleton is not only instrumental in a range of cellular processes including motility, polarity, survival, and replication but is also implicated in the entry, assembly, replication, egress, and spread of innumerable viruses and intracellular bacteria (Taylor et al., 2011). Although pUL135 has the potential to impact on diverse cellular and viral processes, the gene is dispensable in vitro. pUL135 is an extremely effective NK and T cell evasion function, which operates against the background of a number of other HCMV encoded immune evasins. Orchestrated reorganization of the actin cytoskeleton is critical within the cytotoxic cell to promote adherence to the target cell, polarization of cytolytic granules, organization of the immunological synapse, and degranulation. The nucleation-promoting factor WASp and the Arp2/Arp3 complex are recruited to the NK cell synapse (Butler and Cooper, 2009; Mace et al., 2010; Orange, 2008; Rak et al., 2011), whereas actin polymerization induced by WASp and WAVE2 are critical for T cell activation and IS formation (Billadeau et al., 2007). F-actin accumulation at the synapse is a prerequisite for cytotoxic synapses and is required for granule secretion, whereas inhibitory NK synapses are characterized by a lack of an actin ring assembly (Brown et al., 2011; Orange, 2008; Rak et al., 2011). However, there is surprisingly little information regarding the role of actin in target cells. Our imaging studies imply that F-actin fibers provide a framework for the IS. This foundation is undermined in pUL135-expressing cells and correlated with pUL135's capacity to modify F-actin through its interaction with the WRC. A role for the cytoskeleton is indicated both by pUL135's requirement for ABI1/ABI2 and CYFIP1, and pUL135's inability to suppress NK

Figure 4. UL135 Interacts with the WAVE2 Complex and Talin

(A) HFFF-hCAR were infected with RAd-UL135 or RAd-Ctrl, and HFFF were infected with HCMV strain Merlin or MerlinΔUL135. Samples were lysed 48 hr postinfection, and immunoprecipitation was performed for the V5 tag on UL135. Proteins were separated by SDS-PAGE, and western blot was performed for the indicated proteins.

(B–I) HFFF-hCAR were infected with RAd-UL135 or RAd-Ctrl (B, D, F, and H), and HFFF were infected with HCMV strain Merlin or MerlinΔUL135 or mock infected (C, E, G, and I). Samples were fixed and stained for UL135 (V5 antibody) and ABI1 (B and C), WAVE2 (D and E), or talin (H and I) 48 hr postinfection. Samples were lysed, and SDS-PAGE was performed 48 hr postinfection (F) or at the indicated time points (G) followed by western blot for the indicated proteins.

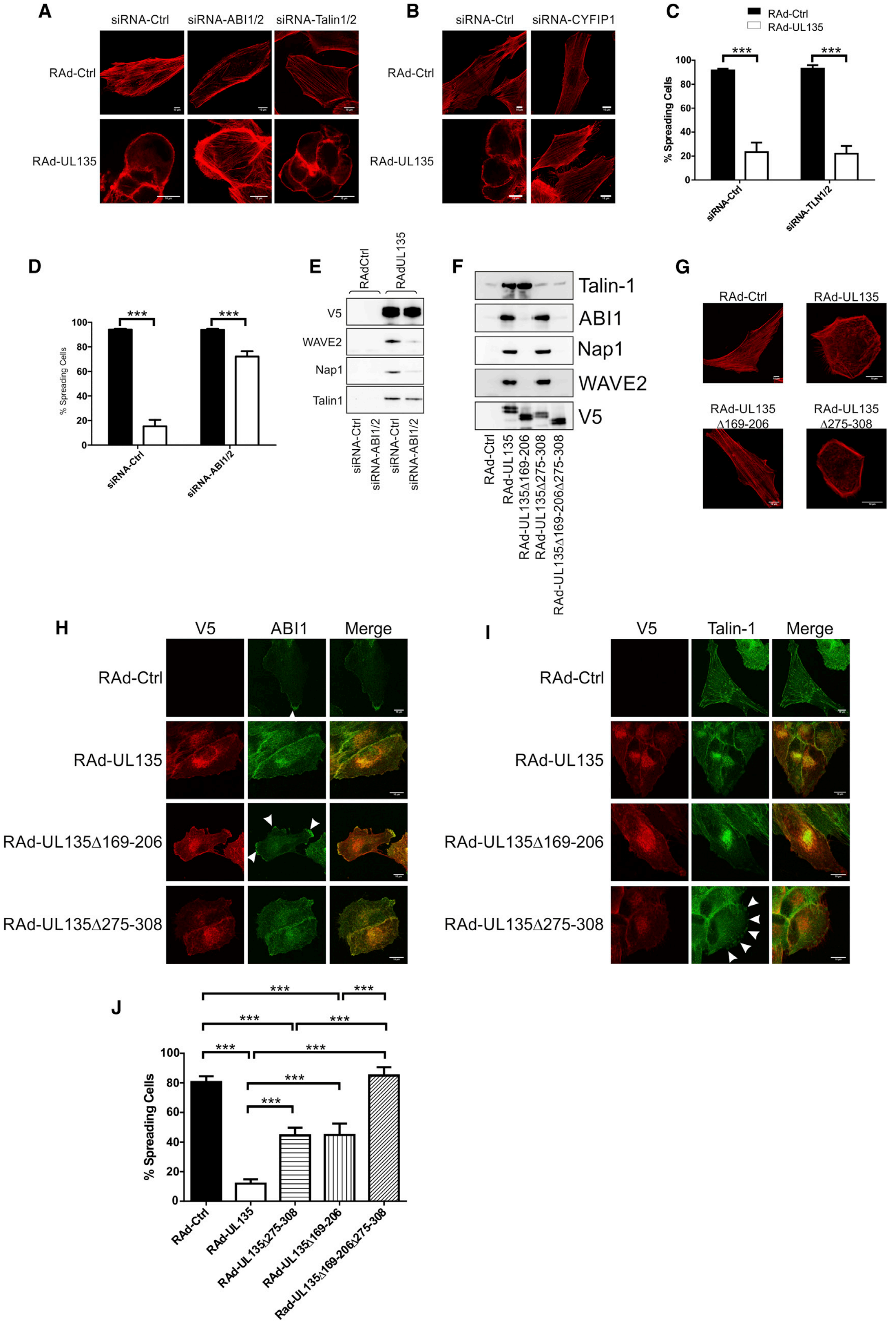

(legend on next page)

cell function in the presence of LatA or jasplakinolide. Neither knockdown nor overexpression of ABI1/ABI2 or CYFIP1 in target cells impacted on NK cell activation, indicating that the interaction of pUL135 with the WRC involves more than simple inhibition of WRC function.

When expressed in isolation, pUL135-mediated disruption of F-actin was associated with the elimination of lamellipodia, filopodia, and nanotubes; by itself, this could reduce recruitment of the cytotoxic cell by the target (Davis and Sowinski, 2008). Within the IS, the highly structured supramolecular activation cluster spatially aligns receptors and adhesion molecules on the effector cell with their ligands on the target cell. The actin cytoskeleton underlies and links to these structures in the target cell, and disruption of actin in the target has been shown to impair ligand binding to receptors on the NK cell (Gross et al., 2010). Formation of the IS was clearly impeded by pUL135, and, when found, they were misformed and failed to induce the prominent actin ring structure characteristic of an activating synapse. By imaging the actin organization at the IS in both the NK and target cell, it was clear that the actin organization within the target cell can influence synapse formation by the NK cell by both delineating the overall extent of the IS and influencing local levels of actin polymerization on a subsynaptic scale. The important role played by target cell F-actin in immune cell recognition is manifest by the remarkable efficiency with which pUL135 suppressed NK and T cell recognition. By preventing the establishment of a cytotoxic synapse, the viral evasion function suppressed a wide range of effector cell types and functions. This mechanism may not be unique to HCMV, given that a wide range of viruses are known to perturb the actin cytoskeleton (Taylor et al., 2011).

EXPERIMENTAL PROCEDURES

Healthy adult volunteers provided blood and dermal fibroblasts following written informed consent. All procedures were approved by the Cardiff University School of Medicine Ethics Committee.

Cells and Viruses

Telomerase-immortalized primary SFs, HFFF, HFFF-hCAR, MRC-5, and 293-TREx cells were grown in Dulbecco's modified Eagle's medium (DMEM; 10% fetal calf serum [FCS]), and primary NK cells were cultured as described previously (Morris et al., 2005; Prod'homme et al., 2007). HCMV IE1- and pp65-specific T cell lines were expanded by coculture with irradiated peptide-coated autologous fibroblasts in RPMI medium (10% FCS, 2% human AB serum, and 10 IU/ml IL-2). The HPV16 E6 T cell clone 7E7 has been described previously (Evans et al., 2001). F-actin binding lifeact peptide (Riedl et al., 2008) tagged with mCherry or mCitrine was expressed from retrovirus vectors. HCMV was derived from bacterial-artificial-chromosome-cloned strain Merlin (RL13^{-} and UL128^{-}) genome (Stanton et al., 2010). Merlin-ΔUL135 was constructed via recombineering in order to delete the first 687 bp of *UL135*. All virus stocks were sequenced (MiSeq) following reconstitution. Recombinant adenoviruses were generated with the AdZ system (Stanton et al., 2008).

Immunohistochemistry

Immunofluorescence and western transfers were performed as described previously (Stanton et al., 2010). For HCMV-infected cells, coverslips were precoated with 10 μg/ml fibronectin in order to provide greater adhesion. There was no difference in the ability of Merlin or Merlin-ΔUL135 to bind to fibronectin (data not shown). Coverslips were blocked with human serum prior to applying primary antibody. For detection of immune synapses, NKL cells were added 15 min before fixation followed by staining with Phalloidin Atto-647N. Images were analyzed with ImageJ (National Institutes of Health).

Immunoprecipitation and Yeast Two-Hybrid

Cells were lysed with IP Lysis Buffer (Pierce Biotechnology) containing 2 mM Na_3VO_4, 2 mM NaF, and protease inhibitors (Sigma-Aldrich) for 30 min and centrifuged at 12,000 × *g* for 30 min, and then 25 μl anti-V5 agarose (Abcam) was added. Samples were rotated for 3 hr and washed three times, and then proteins were eluted in NuPAGE SDS sample buffer. Samples were subjected to SDS-PAGE followed by either western transfer or SILAC mass spectroscopy as previously described (Weekes et al., 2012). Yeast two-hybrid screening was performed as described previously (Striebinger et al., 2013).

siRNA

Cells were transfected with Lipofectamine RNAiMAX (Invitrogen) according to manufacturers' instructions. Cells were infected with RAd or HCMV 24 hr posttransfection and then assayed 48 hr postinfection.

Adhesion and Spreading Assays

Target and NK cells were incubated for 15 min at 37°C in 1 μg/ml CFDA-SE. Cell Tracer or 0.25 μg/ml DDAO-SE (Invitrogen) were used, respectively. Cells were washed and incubated at 37°C for 30 min. Equal quantities of NK and target cells were mixed and incubated at 37°C for 10 min. Cells were fixed in 4% paraformaldehyde before fluorescence-activated cell sorting acquisition. Spreading assays were performed as described previously (Humphries, 2009).

Figure 5. UL135 Interacts Independently with the WRC and Talin and Inhibits Cytoskeletal Remodelling

(A–E) HFFF-hCAR were transfected with control siRNA (siRNA-Ctrl), siRNA against ABI1 and ABI2 (siRNA-ABI1/ABI2), siRNA against talin-1 and talin-2 (siRNA-TLN1/2), or siRNA against CYFIP1. They were infected with RAd-UL135 or RAd-Ctrl 24 hr later. Assays were performed 48 hr postinfection.

(A and B) Cells were fixed and stained for actin (phalloidin).

(C and D) Cells were allowed to adhere to fibronectin-coated dishes for 30 min and then fixed, and the number of cells exhibiting a spread morphology was counted by microscopy. Four separate fields were counted; results are mean ± SD and representative of three independent experiments. Two-way ANOVA test. ***$p < 0.001$.

(E) Cells were lysed, and UL135 was immunoprecipitated with its V5 tag. Immunoprecipitated proteins were separated by SDS-PAGE, and western blot was performed for the indicated proteins.

(F–J) HFFF-hCAR were infected with the indicated adenovirus vectors, and assays were performed at 48 hr postinfection.

(F) UL135 was immunoprecipitated with the V5 tag. Immunoprecipitated proteins were separated by SDS-PAGE, and western blot was performed for the indicated proteins.

(G) Cells were fixed and stained for actin (phalloidin).

(H) Cells were fixed and stained for UL135 (V5 antibody) and ABI1. Accumulation of ABI1 at sites of actin protrusions are indicated with arrows.

(I) Cells were stained for UL135 (V5 antibody) and talin. Accumulation of talin at sites resembling focal adhesions are indicated with arrows.

(J) Cells were allowed to adhere to fibronectin-coated dishes for 30 min and then fixed, and the number of cells exhibiting a spread morphology was counted by microscopy. Four separate fields were counted; results are mean ± SD and are representative of three independent experiments. One-way ANOVA test. ***$p < 0.001$.

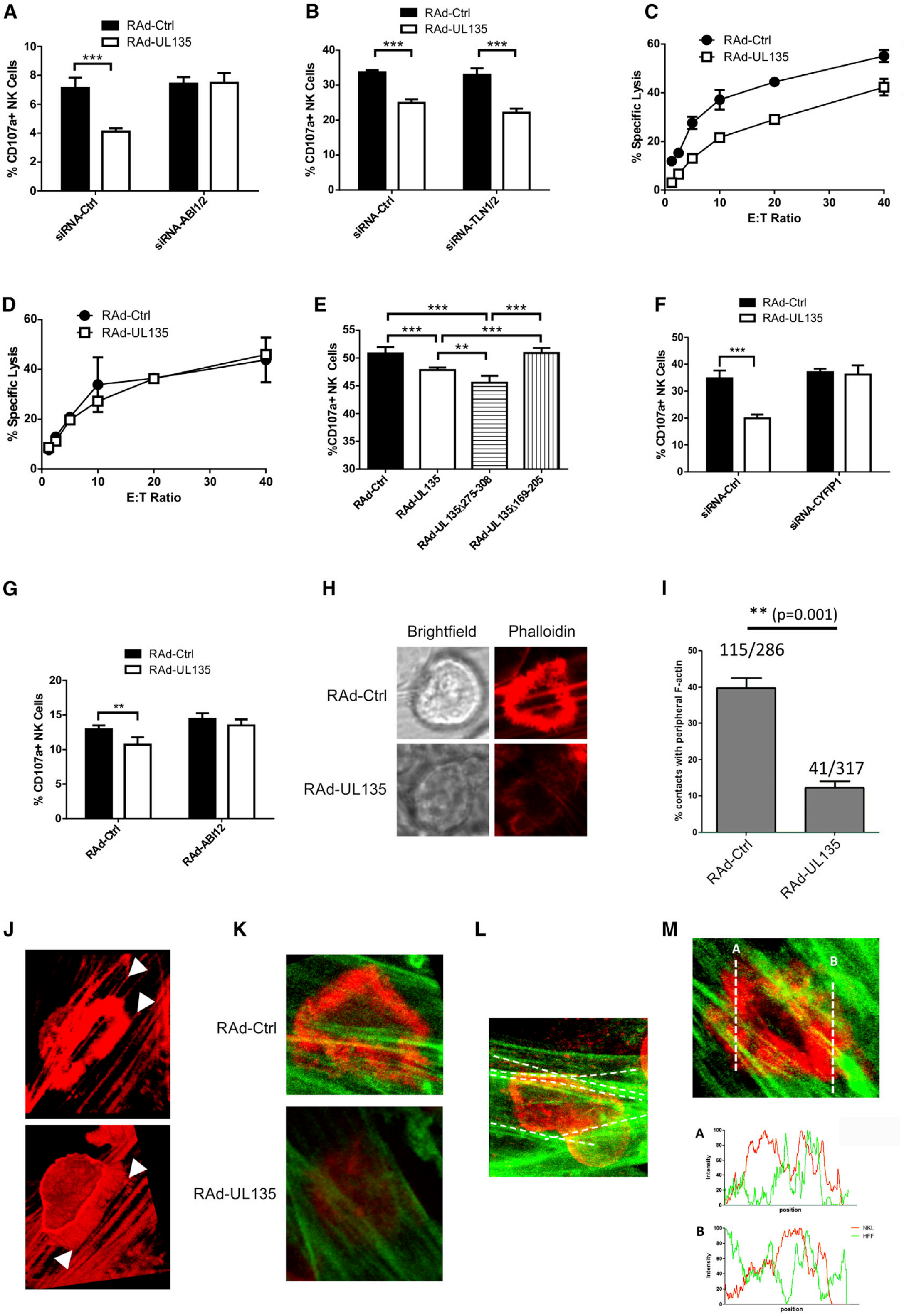

Figure 6. Interactions with ABI1/ABI2 Are Required for UL135 to Protect Against NK Cells

(A and B) HFFF-hCAR were transfected with control siRNA (siRNA-Ctrl), siRNA against ABI1 and ABI2 (siRNA-ABI1/ABI2), or siRNA against talin-1 and talin-2 (siRNA-TLN1/2). They were infected 24 hr later with RAd-UL135 or RAd-Ctrl. NK degranulation assays were performed 48 hr postinfection with IFN-α-activated PBMC.

(legend continued on next page)

Functional Effector Cell Assays

NK cytotoxicity was measured with Cr^{51} release (Aicheler and Stanton, 2013). Degranulation assays were performed as described previously (Prod'homme et al., 2007). For 7E7 T cell stimulation, HLA-A*0201 MRC-5 targets were incubated for 1 hr at 37°C with HPV16 $E6_{29-38}$ peptide (TIHDIILECV). For T cell lines generated from donor D7, autologous fibroblasts were coated with IE1 (VLEETSVML), pp65 (NLVPMVATV), or irrelevant peptide at 100 μg/ml. For IFN-γ assays, cells were stained with combinations of anti-CD3-PerCP, anti-CD8-APC, or anti-CD56-APC before fixation, permeabilization, and intracellular cytokine staining with anti-IFN-γ-FITC or anti-IFN-γ-PE (BD Biosciences). For assays in the presence of LatA and jasplakinolide, cells were maintained in the drug until the CD107a assay. Cells were washed, PBMC was added, and the assay performed. Limited recovery of actin polymerization occurred during the CD107a assay following LatA treatment (Figures S7A and S7B), and none occurred following jasplakinolide treatment (Figure S7C).

SUPPLEMENTAL INFORMATION

Supplemental Information contains Supplemental Experimental Procedures, seven figures, and two tables and can be found with this article online at http://dx.doi.org/10.1016/j.chom.2014.07.005.

AUTHOR CONTRIBUTIONS

R.J.S., V.P., M.A.P., E.C.Y.W., and P.T. performed the experiments. V.P. and M.M. mapped UL135 function. E.C.Y.W., K.L.M., and S.M. performed T cell assays. M.A.P. designed, performed, and analyzed immunological synapse assays. M.H., S.M.B., and J.H. performed yeast two-hybrid. R.A., M.P.W., and P.J.L. performed proteomic analysis. G.S.W. and A.J.D. sequenced HCMV mutants. B.V. generated UL135 antibody. R.A., V.P., R.J.S., M.M., and P.T. performed NK assays. R.J.S. analyzed the interaction with the WRC. R.J.S., V.P., and G.W.G.W. designed the study. R.J.S., V.P., P.T., and G.W.G.W. wrote the manuscript. G.W.G.W. oversaw the project.

ACKNOWLEDGMENTS

This work was supported by funds from the Wellcome Trust WT090323MA and WT093465MA (M.A.P.) and MRC G1000236 and MR/L008734/1. B.V. was supported by projects RECAMO CZ.1.05/2.1.00/03.0101 and GACR P206/12/G151. The authors are grateful to Brett Kaiser, Siân Llewellyn-Lacey, Garry Dolton, Colleen Ring, Anne-Marie Pendergast, Andrea DiSanza, Giorgio Scita, Justin Gundelach, Richard Bram, Daniel D. Billadeau, Susan Craig, and Martin Humphries for technical support, advice, and reagents.

Received: September 14, 2013
Revised: December 10, 2013
Accepted: July 4, 2014
Published: August 31, 2014

REFERENCES

Aicheler, R.J., and Stanton, R.J. (2013). Functional NK cell cytotoxicity assays against virus infected cells. Methods Mol. Biol. *1064*, 275–287.

Arnon, T.I., Achdout, H., Levi, O., Markel, G., Saleh, N., Katz, G., Gazit, R., Gonen-Gross, T., Hanna, J., Nahari, E., et al. (2005). Inhibition of the NKp30 activating receptor by pp65 of human cytomegalovirus. Nat. Immunol. *6*, 515–523.

Benedict, C.A., Butrovich, K.D., Lurain, N.S., Corbeil, J., Rooney, I., Schneider, P., Tschopp, J., and Ware, C.F. (1999). Cutting edge: a novel viral TNF receptor superfamily member in virulent strains of human cytomegalovirus. J. Immunol. *162*, 6967–6970.

Bentz, G.L., Jarquin-Pardo, M., Chan, G., Smith, M.S., Sinzger, C., and Yurochko, A.D. (2006). Human cytomegalovirus (HCMV) infection of endothelial cells promotes naive monocyte extravasation and transfer of productive virus to enhance hematogenous dissemination of HCMV. J. Virol. *80*, 11539–11555.

Billadeau, D.D., Nolz, J.C., and Gomez, T.S. (2007). Regulation of T-cell activation by the cytoskeleton. Nat. Rev. Immunol. *7*, 131–143.

Brown, A.C., Oddos, S., Dobbie, I.M., Alakoskela, J.M., Parton, R.M., Eissmann, P., Neil, M.A., Dunsby, C., French, P.M., Davis, I., and Davis, D.M. (2011). Remodelling of cortical actin where lytic granules dock at natural killer cell immune synapses revealed by super-resolution microscopy. PLoS Biol. *9*, e1001152.

Butler, B., and Cooper, J.A. (2009). Distinct roles for the actin nucleators Arp2/3 and hDia1 during NK-mediated cytotoxicity. Curr. Biol. *19*, 1886–1896.

Cerboni, C., Mousavi-Jazi, M., Linde, A., Söderström, K., Brytting, M., Wahren, B., Kärre, K., and Carbone, E. (2000). Human cytomegalovirus strain-dependent changes in NK cell recognition of infected fibroblasts. J. Immunol. *164*, 4775–4782.

Chapman, T.L., Heikeman, A.P., and Bjorkman, P.J. (1999). The inhibitory receptor LIR-1 uses a common binding interaction to recognize class I MHC molecules and the viral homolog UL18. Immunity *11*, 603–613.

Dargan, D.J., Douglas, E., Cunningham, C., Jamieson, F., Stanton, R.J., Baluchova, K., McSharry, B.P., Tomasec, P., Emery, V.C., Percivalle, E., et al. (2010). Sequential mutations associated with adaptation of human cytomegalovirus to growth in cell culture. J. Gen. Virol. *91*, 1535–1546.

Davis, D.M., and Sowinski, S. (2008). Membrane nanotubes: dynamic long-distance connections between animal cells. Nat. Rev. Mol. Cell Biol. *9*, 431–436.

Evans, M., Borysiewicz, L.K., Evans, A.S., Rowe, M., Jones, M., Gileadi, U., Cerundolo, V., and Man, S. (2001). Antigen processing defects in cervical carcinomas limit the presentation of a CTL epitope from human papillomavirus 16 E6. J. Immunol. *167*, 5420–5428.

Fielding, C.A., Aicheler, R., Stanton, R.J., Wang, E.C., Han, S., Seirafian, S., Davies, J., McSharry, B.P., Weekes, M.P., Antrobus, P.R., et al. (2014).

(C and D) Cytotoxicity assays were performed with IFN-α-activated T-cell-depleted PBMC against HFFF-hCAR transfected with siRNA-Ctrl (C) or siRNA-ABI1/ABI2 (D) and then infected with either RAd-Ctrl or RAd-UL135.
(E) HFFF-hCAR were infected with the indicated adenovirus vectors, and NK cell degranulation assays were performed 48 hr postinfection with IFN-α-activated PBMC.
(F) Cells were transfected with control siRNA or siRNA against CYFIP1. They were infected 24 hr later with RAd-UL135 or RAd-Ctrl. NK degranulation assays were performed 48 hr postinfection with IFN-α-activated PBMC.
(G) HFFF-hCAR were infected with RAd expressing ABI1 and ABI2 (RAd-ABI1+ABI2), RAd-UL135, or empty control vector (RAd-Ctrl) in the combinations indicated, and NK cell degranulation assays were performed 48 hr postinfection with IFN-α-activated PBMC. Results of are means ± SD of quadruplicate samples. Results are representative of three independent experiments. Two-way ANOVA test. $*p < 0.05$, $**p < 0.01$, $***p < 0.001$.
(H and J) HFFF-hCAR were infected with RAd-Ctrl or RAd-UL135. NKL cells were added 48 hr postinfection, allowed to settle, and then fixed and stained with phalloidin. Images show the immune synapse between fibroblasts and NKL cells.
(I) Quantitation of NKL:HFFF interactions in (H).
(J) 3D reconstruction of the IS is shown in the top panel. Arrows indicate actin fibers in the target that define the inner or outer edge of the synapse.
(K and L) Fibroblasts expressing lifeact-Citrine were infected with RAd-Ctrl or RAd-UL135 and incubated with NKL-cell-expressing lifeact-mCherry 48 hr later.
(K) Differential imaging of F-actin within a fibroblast (green) and NKL cell (red) at the IS.
(L) Actin fibers in RAd-Ctrl infected fibroblast that align with the extent of actin polymerization in the NKL cell are highlighted in white.
(M) Intensity profiles of actin along the indicated regions of the IS within the fibroblast (green) or NKL cell (red).

Two novel human cytomegalovirus NK cell evasion functions target MICA for lysosomal degradation. PLoS Pathog. *10*, e1004058.

Gross, C.C., Brzostowski, J.A., Liu, D., and Long, E.O. (2010). Tethering of intercellular adhesion molecule on target cells is required for LFA-1-dependent NK cell adhesion and granule polarization. J. Immunol. *185*, 2918–2926.

Humphries, M.J. (2009). Cell adhesion assays. Methods Mol. Biol. *522*, 203–210.

Kim, C., Ye, F., and Ginsberg, M.H. (2011). Regulation of integrin activation. Annu. Rev. Cell Dev. Biol. *27*, 321–345.

Le, V.T., Trilling, M., and Hengel, H. (2011). The cytomegaloviral protein pUL138 acts as potentiator of tumor necrosis factor (TNF) receptor 1 surface density to enhance ULb'-encoded modulation of TNF-α signaling. J. Virol. *85*, 13260–13270.

Mace, E.M., Zhang, J., Siminovitch, K.A., and Takei, F. (2010). Elucidation of the integrin LFA-1-mediated signaling pathway of actin polarization in natural killer cells. Blood *116*, 1272–1279.

Morris, R.J., Chong, L.K., Wilkinson, G.W., and Wang, E.C. (2005). A high-efficiency system of natural killer cell cloning. J. Immunol. Methods *307*, 24–33.

Orange, J.S. (2008). Formation and function of the lytic NK-cell immunological synapse. Nat. Rev. Immunol. *8*, 713–725.

Penfold, M.E., Dairaghi, D.J., Duke, G.M., Saederup, N., Mocarski, E.S., Kemble, G.W., and Schall, T.J. (1999). Cytomegalovirus encodes a potent alpha chemokine. Proc. Natl. Acad. Sci. USA *96*, 9839–9844.

Prod'homme, V., Griffin, C., Aicheler, R.J., Wang, E.C., McSharry, B.P., Rickards, C.R., Stanton, R.J., Borysiewicz, L.K., López-Botet, M., Wilkinson, G.W., and Tomasec, P. (2007). The human cytomegalovirus MHC class I homolog UL18 inhibits LIR-1+ but activates LIR-1- NK cells. J. Immunol. *178*, 4473–4481.

Prod'homme, V., Sugrue, D.M., Stanton, R.J., Nomoto, A., Davies, J., Rickards, C.R., Cochrane, D., Moore, M., Wilkinson, G.W., and Tomasec, P. (2010). Human cytomegalovirus UL141 promotes efficient downregulation of the natural killer cell activating ligand CD112. J. Gen. Virol. *91*, 2034–2039.

Prod'homme, V., Tomasec, P., Cunningham, C., Lemberg, M.K., Stanton, R.J., McSharry, B.P., Wang, E.C., Cuff, S., Martoglio, B., Davison, A.J., et al. (2012). Human cytomegalovirus UL40 signal peptide regulates cell surface expression of the NK cell ligands HLA-E and gpUL18. J. Immunol. *188*, 2794–2804.

Rak, G.D., Mace, E.M., Banerjee, P.P., Svitkina, T., and Orange, J.S. (2011). Natural killer cell lytic granule secretion occurs through a pervasive actin network at the immune synapse. PLoS Biol. *9*, e1001151.

Riedl, J., Crevenna, A.H., Kessenbrock, K., Yu, J.H., Neukirchen, D., Bista, M., Bradke, F., Jenne, D., Holak, T.A., Werb, Z., et al. (2008). Lifeact: a versatile marker to visualize F-actin. Nat. Methods *5*, 605–607.

Ryu, J.R., Echarri, A., Li, R., and Pendergast, A.M. (2009). Regulation of cell-cell adhesion by Abi/Diaphanous complexes. Mol. Cell. Biol. *29*, 1735–1748.

Seo, J.Y., Yaneva, R., Hinson, E.R., and Cresswell, P. (2011). Human cytomegalovirus directly induces the antiviral protein viperin to enhance infectivity. Science *332*, 1093–1097.

Smith, W., Tomasec, P., Aicheler, R., Loewendorf, A., Nemčovičová, I., Wang, E.C., Stanton, R.J., Macauley, M., Norris, P., Willen, L., et al. (2013). Human cytomegalovirus glycoprotein UL141 targets the TRAIL death receptors to thwart host innate antiviral defenses. Cell Host Microbe *13*, 324–335.

Stanton, R.J., McSharry, B.P., Rickards, C.R., Wang, E.C., Tomasec, P., and Wilkinson, G.W. (2007). Cytomegalovirus destruction of focal adhesions revealed in a high-throughput Western blot analysis of cellular protein expression. J. Virol. *81*, 7860–7872.

Stanton, R.J., McSharry, B.P., Armstrong, M., Tomasec, P., and Wilkinson, G.W. (2008). Re-engineering adenovirus vector systems to enable high-throughput analyses of gene function. Biotechniques *45*, 659–662, 664–668.

Stanton, R.J., Baluchova, K., Dargan, D.J., Cunningham, C., Sheehy, O., Seirafian, S., McSharry, B.P., Neale, M.L., Davies, J.A., Tomasec, P., et al. (2010). Reconstruction of the complete human cytomegalovirus genome in a BAC reveals RL13 to be a potent inhibitor of replication. J. Clin. Invest. *120*, 3191–3208.

Striebinger, H., Koegl, M., and Bailer, S.M. (2013). A high-throughput yeast two-hybrid protocol to determine virus-host protein interactions. Methods Mol. Biol. *1064*, 1–15.

Takenawa, T., and Suetsugu, S. (2007). The WASP-WAVE protein network: connecting the membrane to the cytoskeleton. Nat. Rev. Mol. Cell Biol. *8*, 37–48.

Tani, K., Sato, S., Sukezane, T., Kojima, H., Hirose, H., Hanafusa, H., and Shishido, T. (2003). Abl interactor 1 promotes tyrosine 296 phosphorylation of mammalian enabled (Mena) by c-Abl kinase. J. Biol. Chem. *278*, 21685–21692.

Taylor, M.P., Koyuncu, O.O., and Enquist, L.W. (2011). Subversion of the actin cytoskeleton during viral infection. Nat. Rev. Microbiol. *9*, 427–439.

Tomasec, P., Braud, V.M., Rickards, C., Powell, M.B., McSharry, B.P., Gadola, S., Cerundolo, V., Borysiewicz, L.K., McMichael, A.J., and Wilkinson, G.W. (2000). Surface expression of HLA-E, an inhibitor of natural killer cells, enhanced by human cytomegalovirus gpUL40. Science *287*, 1031.

Tomasec, P., Wang, E.C., Davison, A.J., Vojtesek, B., Armstrong, M., Griffin, C., McSharry, B.P., Morris, R.J., Llewellyn-Lacey, S., Rickards, C., et al. (2005). Downregulation of natural killer cell-activating ligand CD155 by human cytomegalovirus UL141. Nat. Immunol. *6*, 181–188.

Umashankar, M., Petrucelli, A., Cicchini, L., Caposio, P., Kreklywich, C.N., Rak, M., Bughio, F., Goldman, D.C., Hamlin, K.L., Nelson, J.A., et al. (2011). A novel human cytomegalovirus locus modulates cell type-specific outcomes of infection. PLoS Pathog. *7*, e1002444.

Wang, E.C., McSharry, B., Retiere, C., Tomasec, P., Williams, S., Borysiewicz, L.K., Braud, V.M., and Wilkinson, G.W. (2002). UL40-mediated NK evasion during productive infection with human cytomegalovirus. Proc. Natl. Acad. Sci. USA *99*, 7570–7575.

Weekes, M.P., Antrobus, R., Talbot, S., Hör, S., Simecek, N., Smith, D.L., Bloor, S., Randow, F., and Lehner, P.J. (2012). Proteomic plasma membrane profiling reveals an essential role for gp96 in the cell surface expression of LDLR family members, including the LDL receptor and LRP6. J. Proteome Res. *11*, 1475–1484.

Wilkinson, G.W., Tomasec, P., Stanton, R.J., Armstrong, M., Prod'homme, V., Aicheler, R., McSharry, B.P., Rickards, C.R., Cochrane, D., Llewellyn-Lacey, S., et al. (2008). Modulation of natural killer cells by human cytomegalovirus. J. Clin. Virol. *41*, 206–212.

Cell Host & Microbe, Volume *16*

Supplemental Information

HCMV pUL135 Remodels the Actin Cytoskeleton to Impair Immune Recognition of Infected Cells

Richard J. Stanton, Virginie Prod'homme, Marco A. Purbhoo, Melanie Moore, Rebecca J. Aicheler, Marcus Heinzmann, Susanne M. Bailer, Jürgen Haas, Robin Antrobus, Michael P. Weekes, Paul J. Lehner, B. Vojtesek, Kelly L. Miners, Stephen Man, Gavin S. Wilkie, Andrew J. Davison, Eddie C.Y. Wang, Peter Tomasec, and Gavin W.G. Wilkinson

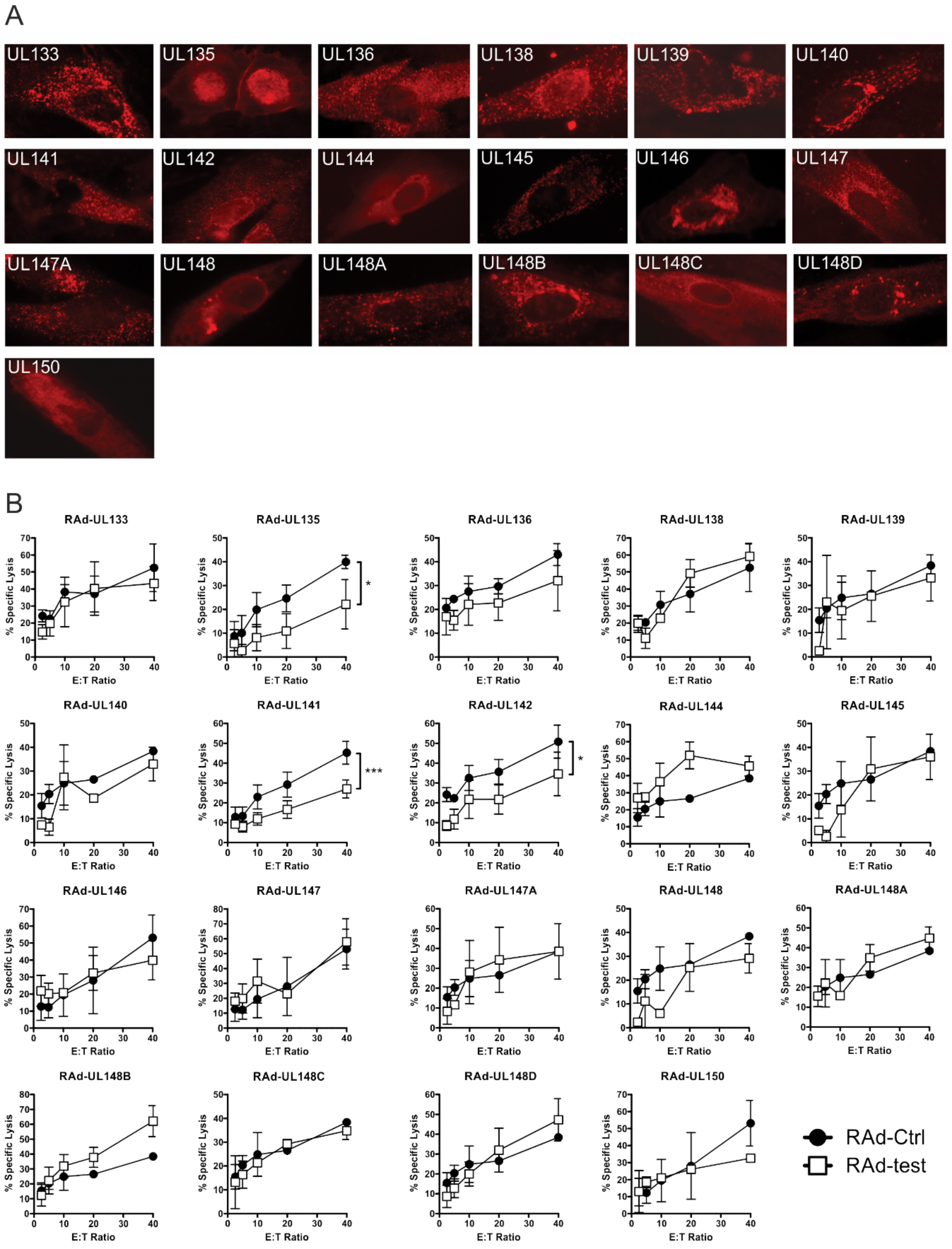

Figure S1, related to Figure 1. Expression, and ability of UL/*b'* proteins to protect from NK cell lysis. (A) HFFF-hCAR were infected with a RAd expressing one of the 19 UL/*b'* genes. 72 h post-infection expression of each gene was tested by immunofluorescence using a C-terminal tag engineered onto each protein. (B) Cytotoxicity assays were set up with the NKL clone against HFFF-hCAR infected with RAd vectors expressing each of the 19 UL/*b'* genes

(RAd-test) or empty vector control (Rad-Ctrl). Assays were performed 72 h after infection. Results are mean ± SD of triplicate samples (Two-Way ANOVA test: * $p < 0.05$, *** $p < 0.001$).

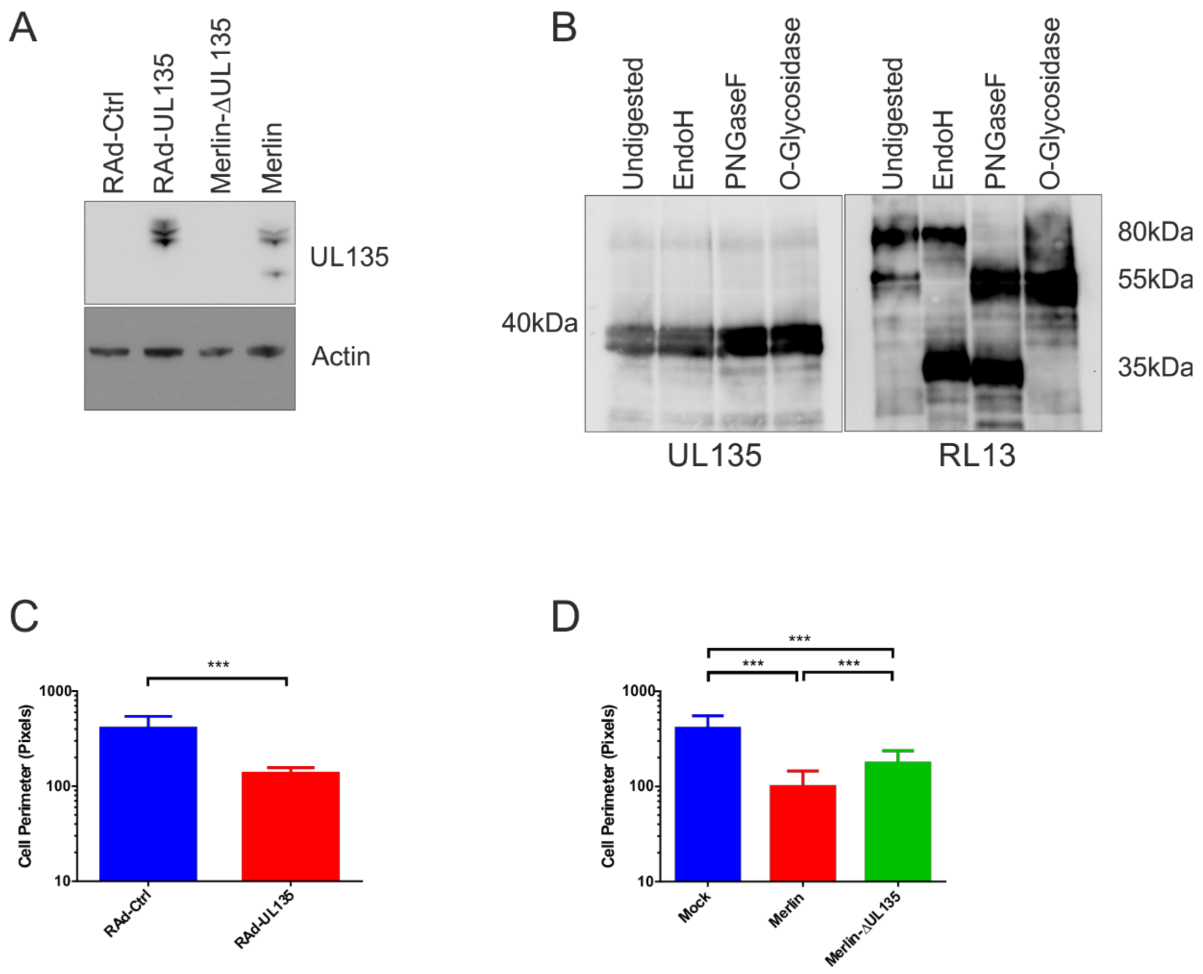

Figure S2, Related to Figure 2. Expression of pUL135. HFFF were infected with Merlin or MerlinΔUL135, and HFFF-hCAR were infected with RAd-Ctrl or RAd-UL135. (A) 48 h post infection samples were separated by SDS-PAGE and western blot performed for the indicated proteins. (B) Prior to SDS-PAGE, samples were deglycosylated with either EndoH, PNGaseF or O-Glycosidase. gpRL13 has both N- and O-linked sugars and therefore provides a positive control for the activity of the enzymes. (C-D) 48 h post infection, the perimeter of cells was calculated using imageJ. Error bars show SD. Unpaired t-test (C) or one-way ANOVA (D): *** $p < 0.001$.

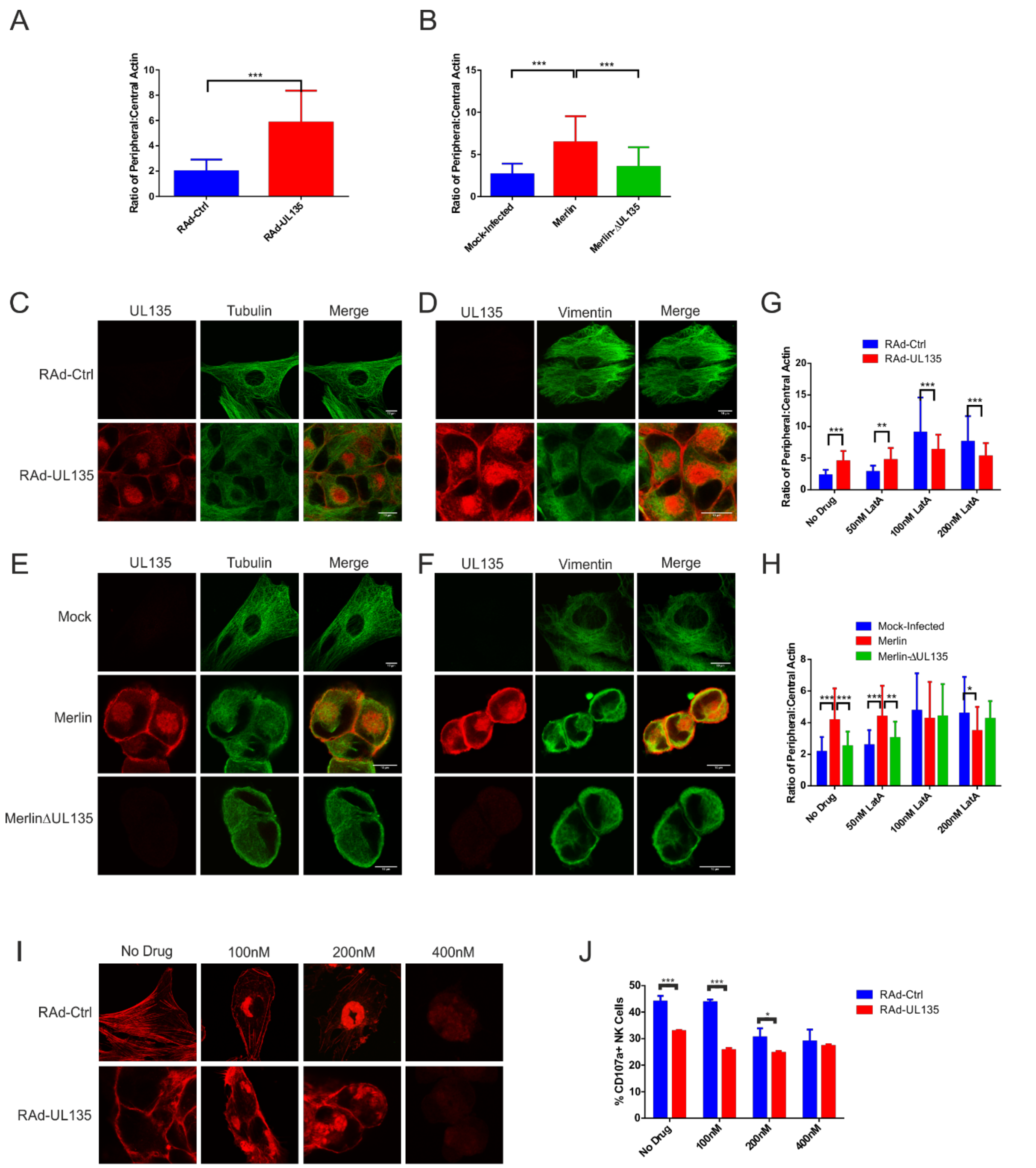

Figure S3, Related to Figure 3. .Effect of pUL135 on the cytoskeleton. HFFF were mock infected, or infected with Merlin or MerlinΔUL135 as indicated. HFFF-hCAR were infected with RAd-Ctrl or RAd-UL135 as indicated. (A) 48 h post infection, actin was stained with phalloidin, and the ratio of actin staining intensity at the cell periphery to staining in the center of the cell calculated. (C-F) 48 h post-infection, samples were stained for UL135 (V5 antibody) and tubulin (C, E) or vimentin (D, F). (G-H) Following infection, cells were treated with latrunculinA at the indicated doses. 48 h post infection, cells were fixed and stained with

phalloidin and the ratio of actin staining intensity at the cell periphery to staining in the center of the cell calculated. (I-J) Following infection, Jasplakinolide was added at the indicated concentrations. 48 hours later, cells were fixed and stained with phalloidin-AF594 (I) or NK degranulation assays were performed with IFNα stimulated PBMC (J). Error bars show SD. Unpaired t-test (A), one-way ANOVA (B), two-way ANOVA (G, H, J): * $p < 0.05$, ** $p<0.01$, *** $p < 0.001$.

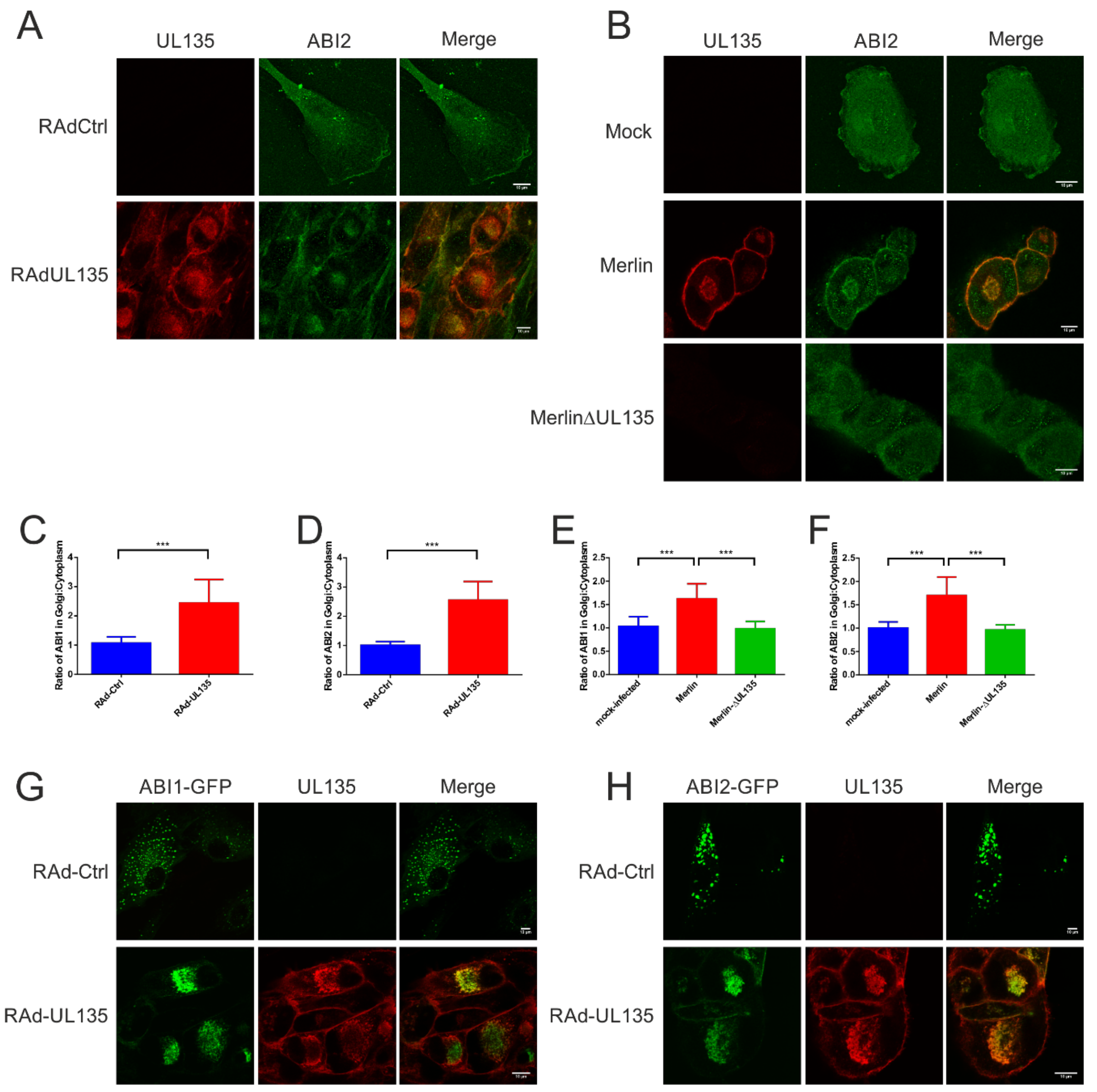

Figure S4, Related to Figure 4. pUL135 interacts with members of the WRC. HFFF-hCAR were infected with RAd-UL135 or RAd-Ctrl (A, C, D, H), and ABI1-GFP (G) or ABI2-GFP (H). HFFF were infected with HCMV strain Merlin, MerlinΔUL135 or mock infected (B, E, F). 48 h post infection samples were fixed and stained for UL135 (V5 antibody), giantin (a marker of the golgi) (C-F) and ABI1 (C, E) or ABI2 (A, B, D, F). (C-F) The amount of ABI1 (C/E) or ABI2 (D/F) that trafficked to the golgi was calculated as the mean fluorescence intensity of ABI1/2 that co-localised with the golgi, divided by the mean fluorescence intensity of ABI1/2 found in the cytoplasm. Error bars show SD. Unpaired t-test (C, D), one-way ANOVA (E, F):* $p < 0.05$, ** $p < 0.01$, *** $p < 0.001$)

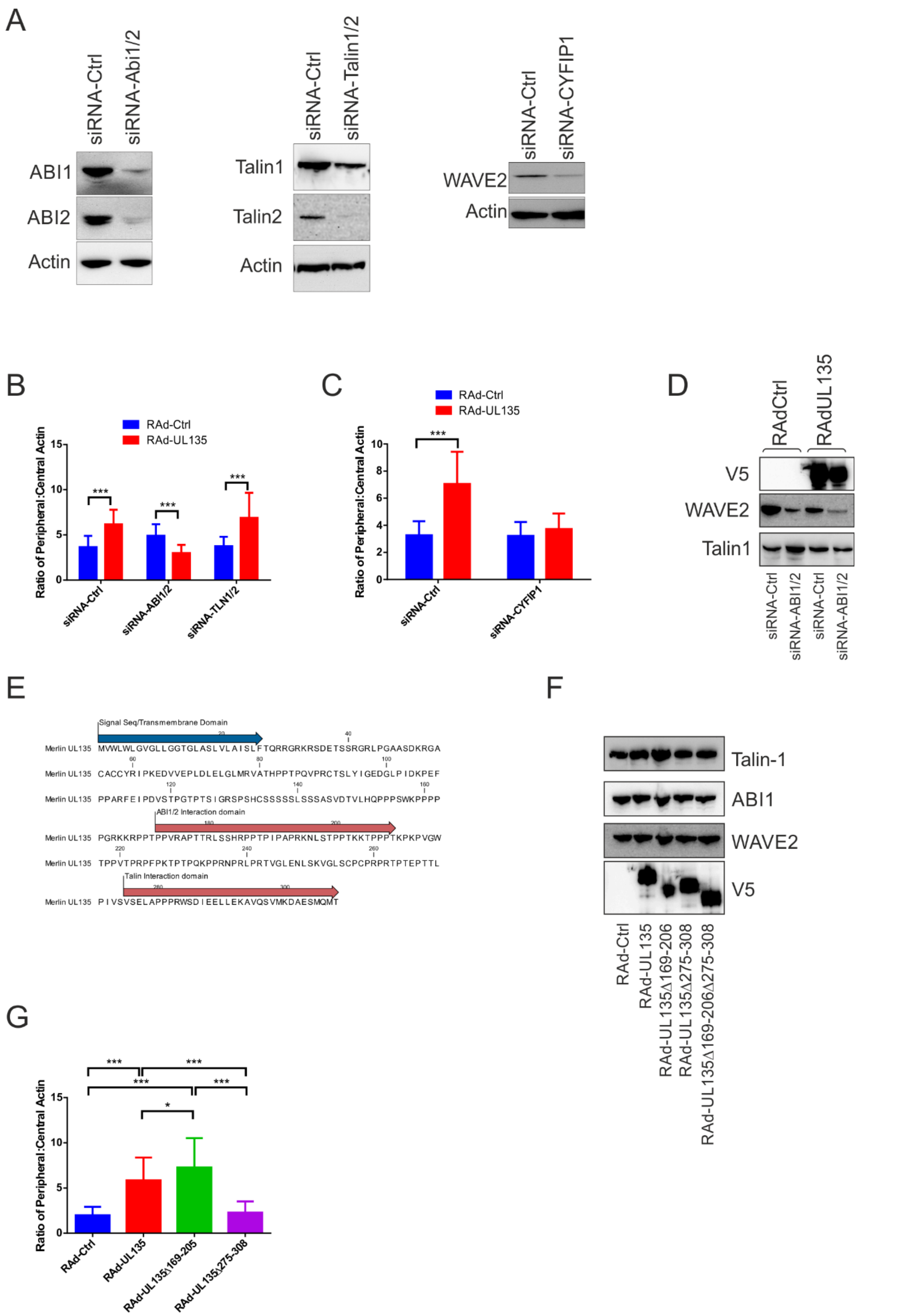

Figure S5, Related to Figure 5. The interaction of UL135 with the WRC results in actin remodelling. (A) Cells were transfected with the indicated siRNA. 72 h post-transfection,

proteins were separated by SDS-PAGE and western blot performed for indicated proteins. Note that for CYFIP1, no antibody was available to detect CYFIP1 in cell lysates, so WAVE2 levels were used as a surrogate marker. (B-D) 24 h after transfection of siRNA, HFFF-hCAR were infected with RAd-Ctrl or RAd-UL135. (B, C) 48 h PI, cells were fixed and stained with phalloidin, then the ratio of staining intensity at the cell periphery to staining in the center of the cell calculated. (D, F) shows input lysates as used in immunoprecipitations (Fig 5E, F). (E) The sequence of the pUL135 is depicted, along with the regions which, which deleted, abrogated the interaction with either the WRC, or Talin. Both a signal sequence (aa 1-22) and transmembrane domain (aa 4-26) are predicted at the N-terminus, indicative of a signal anchor peptide. (G) HFFF-hCAR were infected with the indicated RAd vectors, then 48 h PI, cells were fixed and stained with phalloidin, and the ratio of staining intensity at the cell periphery to staining in the center of the cell calculated. Error bars show SD. One-way ANOVA (G) or two-way ANOVA (B, C): * $p < 0.05$, ** $p < 0.01$, *** $p < 0.001$.

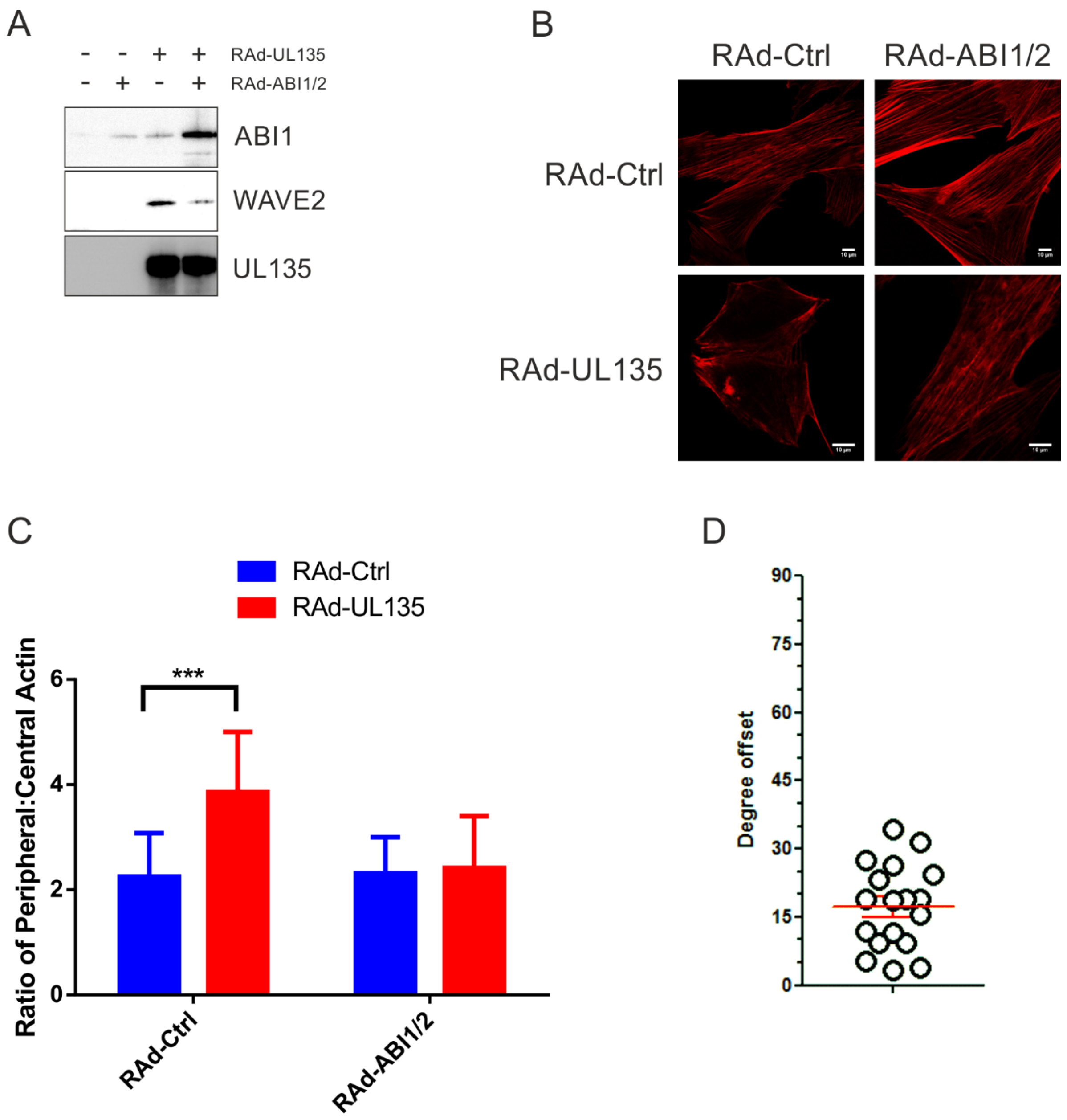

Figure S6, Related to Figure 6. The interaction of pUL135 with the WRC is required for inhibition of NK cells. (A-C) HF-hCAR were infected with either RAd-Ctrl or RAd-UL135, along with either RAd-Ctrl or RAd-ABI1 and RAd-ABI2 together. (A) 48 h post-infection proteins were immunoprecipitated using the V5 tag of UL135, run on a SDS-PAGE gel and western blotted before being stained for the indicated proteins. (B) 48 h post-infection cells were fixed and actin was stained with phalloidin, and (C) the ratio of actin in the periphery of the cell with that in the centre of the cell was calculated. Error bars show SD. Two-way ANOVA test, *** $p < 0.001$. (D) Directionality analysis was performed to determine the preferred orientation of the actin cytoskeleton in the HFF target cell and that of the F-actin in

the NKL cell within individual immune synapses. The plot shows the degree offset between the preferred actin orientation in the NKL cell and that of its target cell, with 0° indicating perfect alignment and 90° indicating a perpendicular alignment. Error bars show SEM. If there were there no correlation between F-actin in the NK synapse and F-actin in the target cell, the values would be equally spread between 0° and 90°. Instead the values cluster, with the NK synapse offset from the actin in the target cell by 15° on average.

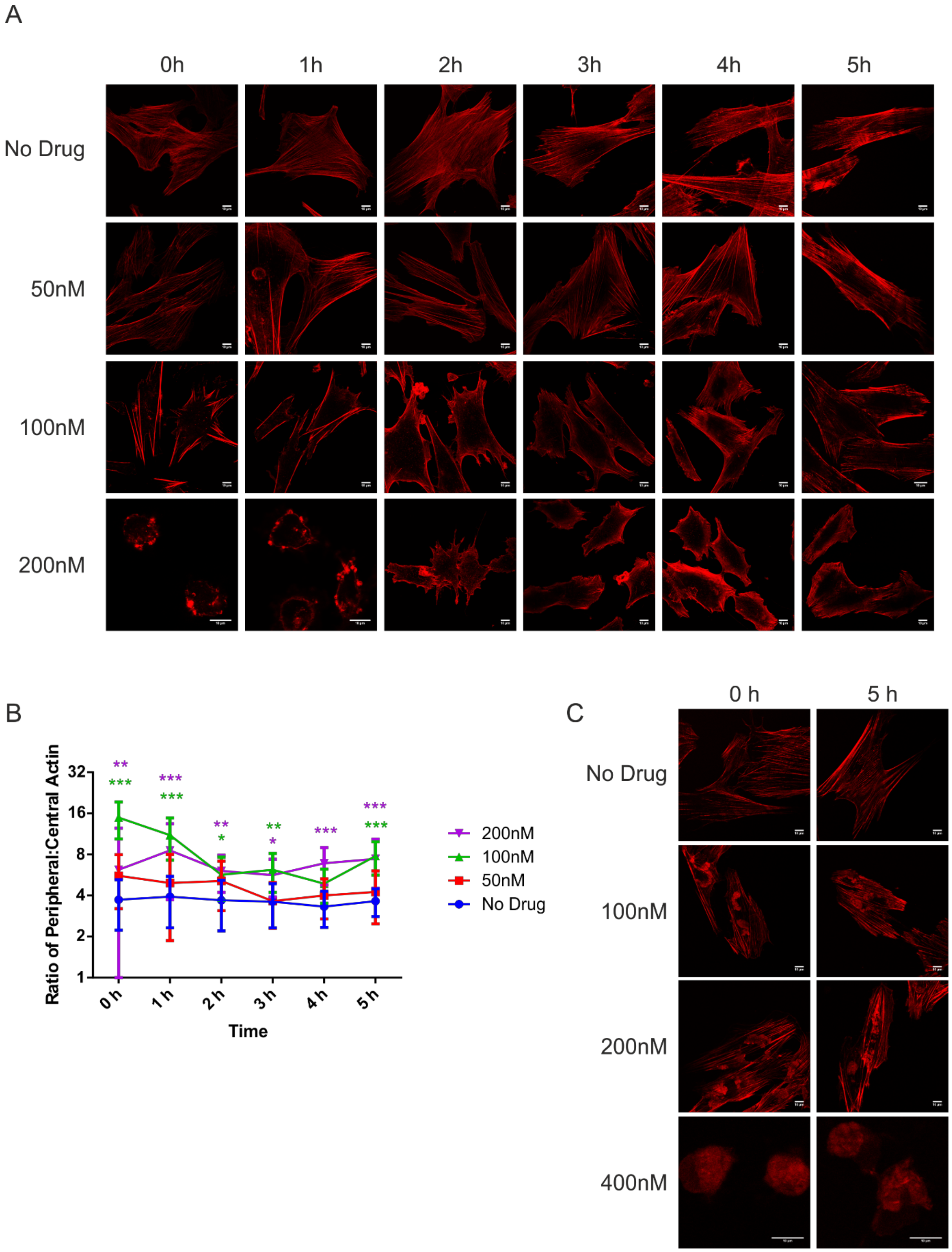

Figure S7, Related to Experimental Procedures. Recovery of actin during the timecourse of a CD107a assay following latrunculin A or jasplakinolide treatment. HFFF-hCAR were treated with LatA (A) or Jasplakinolide (C) for 48 hours, then the drug removed and replaced with RPMI. Cells were allowed to recover for the indicated times, then cells were fixed and

stained with Phalloidin-594. (B) The ratio of central:peripheral actin for the images in (A) were also quantitated. Error bars show SD. Two-way ANOVA, compared to ‘No Drug’: * $p < 0.05$, ** $p < 0.01$, *** $p < 0.001$.

Supplementary Table 1, related to table 4 – Proteins with cell surface expression not modulated by UL135.

NK Receptor Ligands
**MICA-B, ULBP1-5, Nectin 1-2, CD47, CD48, CD66a (CEACAM-1), CD155 (PVR), CD324 (E-Cadherin), Necl2 (IGSF4), NTB-A (SLAMF6), CRACC (SLAMF7), HLA A, B, C, E.**
**Adhesion Proteins**
**CD2, CD11b, CD22, CD29 (integrin β1), CD31, CD34, CD36, CD41, CD42b, CD43, CD44, CD49a (integrin α1), CD49c (integrin α3), CD49d (integrin α4), CD49e (integrin α5), CD49f (integrin α6), CD50, CD51/CD61 (integrin αvβ3), CD54 (ICAM-1), CD55 (DAF), CD56, CD58 (LFA-3), CD99, CD99R, CD147, CD162.**

HFFF-hCAR were infected for 72 h with a replication-deficient adenovirus expressing UL135 or a control adenovirus. Surface stainings were done with specific antibodies and isotype controls. Cells were analyzed by flow cytometry.

Supplementary Table 2, related to Fig. 4 – SILAC immunoprecipitation of UL135 interacting proteins

Protein Names	Uniprot	Log_2 H/L	H/L Sig A	Ratio Count	Unique peptides
NAP-1	Q9Y2A7	-4.620175	8.05E-07	15	8
Sra-1/PIR121	Q7L576	-4.549283	1.20E-06	14	10
UL135	F5HAQ7	-4.405956	2.63E-06	50	10
Talin-1	Q9Y490	-3.766976	6.50E-05	39	23
WAVE-2	Q9Y6W5	-3.696789	8.98E-05	2	3

Identified proteins are shown with corresponding UniProt accession number. SILAC ratios are shown as log_2 non-normalised values with associated significance A values as calculated in Persues v.1.1.1.13. SILAC ratios were calculated on the basis of all identified razor and unique peptides (ratio count) with the number of unique peptides identified for each protein indicated.

Supplementary Experimental Procedures

Generation of recombinant HCMV

To generate MerlinΔUL135 a cassette comprising ampicillin resistance, lacZ and SacB was recombineered in place of nt 189772-189086 (accession no. NC_006273) using primers 5’-CGGTGCGCGGCAGTCTCGGATTCCGCGGTGGCTTTTGTGGCGTCGGCGTTT TCGGGAAGGGCCTGGGCGTCACCGGCGCCTGTGACGGAAGATCACTTCG-3’ and 5’-CGGCCCTTCCCGACACGGAGTTTGAGATTCCAAGCAGGAGAGAAGATCATGGTG TGGCTGTGGCTCGGCGTCGGGCTCCTGAGGTTCTTATGGCTCTTG-3’. The cassette was removed using primer 5’-AGCCCGACGGTGCGCGGCAGTCTCGGATTCCGCGGTGGCTTT TGTGGCGTGATCTTCTCTCCTGCTTGGAATCTCAAACTCCGTGTCGGGAAGGGCC GGT-3’, deleting the first 687bp of the *UL135* open reading frame. MerlinΔUL141 has been described (Prod'homme et al., 2010). MerlinΔUL135/ΔUL141 BAC was generated from MerlinΔUL141 using the same strategy. To optimize detection in the context of a HCMV strain Merlin infection, a sequence providing a C-terminal V5 tag was inserted into *UL135.* The cassette comprising ampicillin resistance, lacZ and SacB was recombineered at the end of *UL135* using primers 5’-TCTCTTAGCTGCGGTGAAAAAGAGGGAAGGCGTGTGCTGCTATACAACTGTACA ACGGACGCGCTCGCTGTTTCGGTCCCTGTGACGGAAGATCACTTCG-3’ and 5’-ATGGTCGGACATCGAGGAACTCTTGGAAAAGGCGGTGCAGAGCGTCATGAAGGA CGCCGAGTCAATGCAGATGACCTGACTGAGGTTCTTATGGCTCTTG-3’. This cassette was then removed using primers 5’-GGGAAGGCGTGTGCTGCTATACAACTGTACAACGGACGCGCTCGCTGTTTCGGTC TTACGTAGAATCAAGACCTAGGAGCGGGTTAGGGATTGGCTTACC-3’ and 5’-AAAGGCGGTGCAGAGCGTCATGAAGGACGCCGAGTCAATGCAGATGACCAGCG CTGGTAAGCCAATCCCTAACCCGCTCCTAGGTCTTGATTCTACGTAA-3’. These primers result in the insertion of an in-frame V5 tag (underlined) at the extreme C-terminus of *UL135*, linked to *UL135* by a Ser-Ala linker. Virus was recovered by electroporating BACs into HFFF using an Amaxa Nucleofector (program T16). In functional assays virus with either native *UL135,* or *UL135* containing a C-terminal V5 tag behaved similarly, however in the results presented, virus containing a native *UL135* was used in functional assays, and virus containing a C-terminal V5 tag was used in western blot and immunofluorescence assays.

High throughput DNA sequencing

Sequence data sets were generated using either a Genome Analyzer IIx (Sir Henry Wellcome Functional Genomics Facility, University of Glasgow) or MiSeq instrument (in house; Illumina, San Diego, CA, USA). 1000ng of DNA from each sample was sheared by sonication and DNA fragments were purified and size-selected using AMPure XP beads (Beckman-Coulter, High Wycombe, UK). Purified DNA fragments were end-repaired, 3'-adenylated, ligated to paired-end multiplexing adapters and amplified by PCR using standard methods (Illumina). Sequence read data sets were filtered to remove bases less than phred quality 30 and processed for adapter removal using Trim Galore v. 0.2.2, which is a wrapper for Cutadapt and FastQC (http://www.bioinformatics.babraham.ac.uk/projects/trim_galore). Filtered reads were aligned against reference genomes using BWA v0.6.2-r126 (Li and Durbin, 2010). Indexed Bam files were generated from the alignments using Samtools v. 0.1.18 (Li et al., 2009) and viewed using Tablet 1.12.12.05 (Milne et al., 2010).

Generation of recombinant adenoviruses

Recombinant adenoviruses (RAd) were generated using the AdZ system (Stanton et al., 2008). Infection efficiency of RAds on all cell types was initially determined using a RAd expressing eGFP, and subsequently by immunofluorescence for the gene of interest. The *UL135* gene was amplified from Merlin DNA, with a C-terminal V5 tag, using primers 5'-AACCGTCAGATCGC CTGGAGACGCCATCCACGCTGTTTGACCTCCATAGAAGACACCGGGACCGATCC AGCCTGGATCCGCAGGAGAGAAGATCATGGT-3' and 5'-ATAGAGTATACAATAGTGACG TGGGATCCTTACGTAGAATCAAGACCTAGGAGCGGGTTAGGGATTGGCTTACCA GCGCTGGTCATCTGCATTGACTCGG-3', or without any tag. Viruses are all referred to as RAd-UL135. NK assays from Figs. 1 and 2 were repeated using virus lacking or containing a tag, and no difference was detected. In other figures, virus with a V5 tag was used.

Deleting the domains of UL135 that are required for interacting with ABI1/2 and talin was performed by recombineering as previously described (Stanton et al., 2010). For deletion of aa275-308 the amp^r/sacB/lacZ cassette was amplified and inserted using primers 5'-TCGGCCAGCGTCGACACGGTACTGCATCAGCCGCCGCCATCCTGGAAGCCACCTC CGCCGCCCGGGCGCAAGAAGCGGCCTGTGACGGAAGATCACTTCG-3' and 5'-

TAGAGTATACAATAGTGACGTGGGATCCTTACGTAGAATCAAGACCTAGGAGCG GGTTAGGGATTGGCTTACCAGCGCTCTGAGGTTCTTATGGCTCTTG-3’, then removed using primer 5’-TATAGAGTATACAATAGTGACGTGGGATCCTTACGTAGAATCAAGACCTAGGAG CGGGTTAGGGATTGGCTTACCAGCGCTCGACACGATAGGCAGCGTG-3’. For deletion of aa169-205 the amp^r/sacB/lacZ cassette was amplified and inserted using primers 5’-TCGGCCAGCGTCGACACGGTACTGCATCAGCCGCCGCCATCCTGGAAGCCACCTC CGCCGCCCGGGCGCAAGAAGCGGCCTGTGACGGAAGATCACTTCG-3’ and 5’-TACGCGGCGCGGGTATCGGCGTCGGGGGCCTGTGCGACGACAGCCGCGTGGTGG GGGCCCGGACCGGCGGCGTAGGCGGCTGAGGTTCTTATGGCTCTTG-3’. Then removed using primer 5’-AGCCGCCGCCATCCTGGAAGCCACCTCCGCCGCCCGGGCGCAAGAAGCGGCCGC CCACGAAACCCAAGCCGGTCGGCTGGACACCGCCGGTGACGCCCAG-3’.

ABI1 was PCR amplified using primers 5’-CGTCAGATCGCCTGGAGACGCCATCCACGC TGTTTTGACCTCCATAGAAGACACCGGGACCGATCCAGCCTGGATCCACCATGGC A GAGCTGCAGATGTT-3’ (untagged construct) or 5’-TGGAGTTCGTGACCGCCGCCGGGA TCACTCTCGGCATGGACGAGCTGTACAAGGGTAGCGCTGGATCAGCAGGGTCCG CGATGGCAGAGCTGCAGATGTT-3’ (N-terminal GFP tag) along with 5’-GTTTATTGAGT AGGATTACAGAGTATAACATAGAGTATAATATAGAGTATACAATAGTGACGTGG GATCCTTAATCAGTATAGTGCATGATTGATTCAAC-3’. ABI-2 was amplified using primers 5’-CGTCAGATCGCCTGGAGACGCCATCCACGCTGTTTTGACCTCCATAGAAGACACC GGGACCGATCCAGCCTGGATCCACCATGTCCTGCAGATGCTGGAT-3’ (untagged construct) or 5’-TGGAGTTCGTGACCGCCGCCGGGATCACTCTCGGCATGGACGAGCTG TACAAGGGTAGCGCTGGATCAGCAGGGTCCGCGATGTCCTGCAGATGCTGGAT-3’ (N-terminal GFP tag) along with 5’-ACGTTTATTGAGTAGGATTACAGAGTATAACATA GAGTATAATATAGAGTATACAATAGTGACGTGGGATCCTTACTCAGAATAATGC ATGATAGACTCA-3’. Templates were pEGFP-C1-Abi1 (a kind gift from Giorgio

Scita/Andrea DiSanza) and pEYFP-Abi2 (a kind gift from Ann-Marie Pendergast) (Courtney et al., 2000).

RAd were recovered by transfecting into 293TREx cells using Effectene (Qiagen), and were subsequently propagated and titrated in 293TREx cells. RAd-UL141 has been described (Tomasec et al., 2005). RAd-Ctrl is a control adenovirus without a transgene insert.

Generation of lifeact expressing cell lines

A DNA sequence encoding the F-actin binding lifeact peptide (Riedl et al., 2008) was generated by annealing of complementary primers 5'-CTAGCGGATCCTAGGCCACCATGGGGGTGG CCGACCTGATCAAGAAGTTCGAGAGCATCAGCAAGGAGGAGCCA-3' and 5'-TGGCTCCTCCTTGCTGATGCTCTCGAACTTCTTGATCAGGTCGGCCACCCCCATGG TGGCCTAGGATCCGCTAG-3'. This sequence was C-terminally tagged with fluorescent proteins by inserting into pcDNA3.1 (Invitrogen, Paisley, UK) encoding mCherry or Citrine fluorescent proteins. Flourescent lifeact constructs were subcloned into a mouse Moloney murine leukaemia virus-based retroviral vector. NKL cells and fibroblasts were stably transfected using the Phoenix retroviral transfection system, with transfectants being selected and maintained in 10 μg/ml blasticidin (Invitrogen).

SILAC

Following in-gel reduction and alkylation, proteins were digested using trypsin with the resulting peptides eluted and dried down for LC-MSMS analysis. LC-MSMS was performed on an OrbiTrap XL coupled to a nanoAcquity. Peptides were resolved using a gradient rising from 7 to 35% acetonitrile by 45 min and 85% acetonitrile by 55 min. MS spectra were acquired at 60,000 resolution (fwhm) with MSMS spectra acquired in the LTQ using a top 6 method and 2 microscans. Samples were acquired in duplicate and raw files processed using MaxQuant v.1.1.1.14. Data were searched, using the embedded search engine Andromeda, against a Uniprot database comprising human and HCMV strain Merlin sequences (downloaded 310112). Carbamidomethyl (C) was set as a fixed modification while oxidised (M), deamidation (NQ) and acetylation (protein N-terminus) were allowed as potential variable modifications. Protein and peptide FDR were set at 0.01. Protein quantitation was based on razor and unique peptides with reported proteins requiring a minimum of 1 ratio count, re-

quantitation was enabled. MaxQuant output was processed in Perseus v.1.1.1.13 to generate significance A values.

Yeast 2 hybrid

IN order to generate a bait strain, the UL135 ORF was amplified using primers 5'-GGCCGGATCCGTATGGTGTGGCT GTGGCTC-3' and 5'-GGCCCTGCAGTCAGGTCATCTGCATTGACT-3' and cloned into BamH1/PstI sites of vector pGBKT7. A yeast bait strain was generated by transforming AH109 cells with this vector. Y187 cells pretransformed with a Human Testis MATCHMAKER cDNA Library (Clontech) were used as prey source.

siRNA

siRNA (Qiagen) were as follows: ABI1 (SI02655338), ABI2 (SI00299075), WAVE2 (SI02664312), talin-1 (SI00086982), talin-2 (SI00109263), CYFIP1 (SI04203717) and Allstars negative control (1027417).

Immunohistochemistry

Primary antibodies were mouse anti-V5 (MCA1360GA, Serotec), anti-ABI1 (a gift from Giorgio Scita (Innocenti et al., 2004)), rabbit anti-V5 (ab9116, Abcam), anti-giantin (ab24586, Abcam), anti-actin (Sigma), anti-WAVE2 (a gift from Dan Billadeau (Nolz et al., 2006)), goat anti-ABI2 (sc-20327, Santa-cruz), anti-NAP1 (N3788, Sigma), anti-talin-1 (TA205, Sigma), anti-talin-2 (121A, abcam), anti-PIR121 (P0092, Sigma) and anti-Vimentin (V6630, Sigma). Secondary antibodies were anti-goat-HRP (Santa-cruz), anti-mouse-HRP and anti-rabbit-HRP (GE Healthcare) or Alexa Fluor 594 and 488 conjugated antibodies (Invitrogen).

Directionality analysis

2 colour Z-stack imaging of NKL/HFFF interfaces was performed to image the actin cytoskeleton either side of the immune synapse. Z-images were collapsed by maximum projection. Directionality of actin fibres in the target cell and the F-actin in at the immune synapse of the NKL cell was determined separately in Fiji/ImageJ using local gradient analysis. The distribution of orientations was fitted to a Gaussian function, the centre of which reports the preferred orientation of the actin cytoskeleton in the respective images.

Supplemental References

Courtney, K.D., Grove, M., Vandongen, H., Vandongen, A., LaMantia, A.S., and Pendergast, A.M. (2000). Localization and phosphorylation of Abl-interactor proteins, Abi-1 and Abi-2, in the developing nervous system. Mol Cell Neurosci *16*, 244-257.

Li, H., and Durbin, R. (2010). Fast and accurate long-read alignment with Burrows-Wheeler transform. Bioinformatics *26*, 589-595.

Li, H., Handsaker, B., Wysoker, A., Fennell, T., Ruan, J., Homer, N., Marth, G., Abecasis, G., Durbin, R., and Genome Project Data Processing, S. (2009). The Sequence Alignment/Map format and SAMtools. Bioinformatics *25*, 2078-2079.

Milne, I., Bayer, M., Cardle, L., Shaw, P., Stephen, G., Wright, F., and Marshall, D. (2010). Tablet--next generation sequence assembly visualization. Bioinformatics *26*, 401-402.

Prod'homme, V., Sugrue, D.M., Stanton, R.J., Nomoto, A., Davies, J., Rickards, C.R., Cochrane, D., Moore, M., Wilkinson, G.W., and Tomasec, P. (2010). Human cytomegalovirus UL141 promotes efficient downregulation of the natural killer cell activating ligand CD112. J Gen Virol *91*, 2034-2039.

Riedl, J., Crevenna, A.H., Kessenbrock, K., Yu, J.H., Neukirchen, D., Bista, M., Bradke, F., Jenne, D., Holak, T.A., Werb, Z., *et al.* (2008). Lifeact: a versatile marker to visualize F-actin. Nat Methods *5*, 605-607.

Stanton, R.J., Baluchova, K., Dargan, D.J., Cunningham, C., Sheehy, O., Seirafian, S., McSharry, B.P., Neale, M.L., Davies, J.A., Tomasec, P., *et al.* (2010). Reconstruction of the complete human cytomegalovirus genome in a BAC reveals RL13 to be a potent inhibitor of replication. J Clin Invest *120*, 3191-3208.

Stanton, R.J., McSharry, B.P., Armstrong, M., Tomasec, P., and Wilkinson, G.W. (2008). Re-engineering adenovirus vector systems to enable high-throughput analyses of gene function. BioTechniques *45*, 659-662, 664-658.

Tomasec, P., Wang, E.C., Davison, A.J., Vojtesek, B., Armstrong, M., Griffin, C., McSharry, B.P., Morris, R.J., Llewellyn-Lacey, S., Rickards, C., *et al.* (2005). Downregulation of natural killer cell-activating ligand CD155 by human cytomegalovirus UL141. Nat Immunol *6*, 181-188.
